# PocketDTA: an advanced multimodal architecture for enhanced prediction of drug−target affinity from 3D structural data of target binding pockets

**DOI:** 10.1093/bioinformatics/btae594

**Published:** 2024-10-04

**Authors:** Long Zhao, Hongmei Wang, Shaoping Shi

**Affiliations:** Department of Mathematics, School of Mathematics and Computer Sciences, Nanchang University, Nanchang 330031, China; Department of Mathematics, School of Mathematics and Computer Sciences, Nanchang University, Nanchang 330031, China; Department of Mathematics, School of Mathematics and Computer Sciences, Nanchang University, Nanchang 330031, China; Institute of Mathematics and Interdisciplinary Sciences, Nanchang University, Nanchang 330031, China

## Abstract

**Motivation:**

Accurately predicting the drug−target binding affinity (DTA) is crucial to drug discovery and repurposing. Although deep learning has been widely used in this field, it still faces challenges with insufficient generalization performance, inadequate use of 3D information, and poor interpretability.

**Results:**

To alleviate these problems, we developed the PocketDTA model. This model enhances the generalization performance by pre-trained models ESM-2 and GraphMVP. It ingeniously handles the first 3 (top-3) target binding pockets and drug 3D information through customized GVP-GNN Layers and GraphMVP-Decoder. In addition, it uses a bilinear attention network to enhance interpretability. Comparative analysis with state-of-the-art (SOTA) methods on the optimized Davis and KIBA datasets reveals that the PocketDTA model exhibits significant performance advantages. Further, ablation studies confirm the effectiveness of the model components, whereas cold-start experiments illustrate its robust generalization capabilities. In particular, the PocketDTA model has shown significant advantages in identifying key drug functional groups and amino acid residues via molecular docking and literature validation, highlighting its strong potential for interpretability.

**Availability and implementation:**

Code and data are available at: https://github.com/zhaolongNCU/PocketDTA.

## 1 Introduction

Drug−target interaction (DTI) (Tapio *et al.* 2015) studies play a central role in the overall process of drug discovery. DTA can be used to analyze the strength of DTI. Commonly used traditional computational methods such as molecular docking (Guedes *et al.* 2014) and molecular dynamics simulation (Naqvi *et al.* 2018) can accurately predict affinity values, but are time-consuming and labor-intensive in practical applications (Kairys *et al.* 2019). In contrast, machine learning and deep learning techniques show significant speed advantages in DTA prediction. Particularly, deep learning methods show excellent potential for recognizing complex bioinformatic patterns and extracting key features (Dhakal *et al.* 2022).

In recent years, DTA prediction models based on deep learning have been continuously developed and some efficient methods have emerged. Initially, Öztürk *et al.* (2018) introduced DeepDTA, the first convolutional neural network (CNN) model to learn representations from ligand SMILES and protein sequences, predicting DTA with promising results. To further incorporate more sequence information, Öztürk *et al.* (2019) introduced WideDTA. In contrast, Nguyen *et al.* (2020) argued that string-based drug representations are less natural, favoring graph models. Following this, they introduced GraphDTA, using graphs to represent drugs, processed with graph convolutional network (GCN). Results indicate that the GraphDTA model outperforms previous models but disregards protein structure. Subsequently, DGraphDTA (Jiang *et al.* 2020) further progressed by mapping proteins into 2D graphs and gained structural insights via GCN. Moreover, He *et al.* (2023) noted that sequence-based algorithms fail to capture structural information, whereas graph-based algorithms lack in feature extraction and interaction. This led to the proposal of NHGNN-DTA, a hybrid network designed to capture drug and protein features as 2D graphs, thereby enhancing information interaction. This approach combines sequence and graph methods for DTA prediction. Recently, 3DprotDTA (Voitsitskyi *et al.* 2023) represented a novel attempt that uses AlphaFold to predict the 3D structure of proteins and used GCN to transform this structure into a 2D graphical representation for learning.

While these deep learning-based methods achieve rapid DTA prediction, they exhibit several limitations. Firstly, the generalization performance is insufficient. The inputs of DTA in real-world application scenarios exhibit a significant diversity, yet the drug and target representations for most methods rely exclusively on DTA benchmark datasets. The reliance on benchmark datasets inherently constrains the generalization capabilities of these methods when applied to unfamiliar data. Second, the utilization of 3D information remains insufficient. The nature of drug−target binding involves physicochemical interactions between small molecules and macromolecules in 3D space. Nevertheless, the existing methods predominantly depend on 1D sequences and 2D graphical representations of the drug and target (Jiang *et al.* 2022, Gu *et al.* 2023, Peng *et al.* 2024, Tian *et al.* 2024), neglecting the examination of 3D spatial aspects of the binding process. Lastly, the interpretability is poor. DTI is essentially an interaction between crucial drug functional groups and pivotal amino acid residues within target binding pockets. Yet, many previous methods have overlooked the direct investigation of these localized interactions (Abbasi *et al.* 2020, Zheng *et al.* 2020, Yuan *et al.* 2022, Zhou *et al.* 2024). As a result, it is difficult to explain the specific mechanism of DTI in depth, which is a significant shortcoming in the drug design and optimization process.

**Figure 1.**
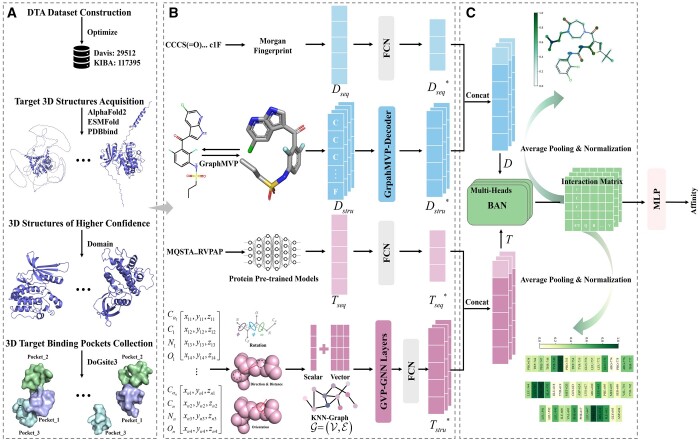
The overall flow of the PocketDTA. (A) Data preprocessing module: includes steps such as updating target sequences, removing redundant samples, obtaining 3D coordinates of targets, extracting high-confidence protein domains for precise 3D coordinates, and obtaining the target binding pocket. (B) Multimodal representation and feature extraction module: involves the sequence and structural characterization of drugs and targets, followed by specific feature extraction processes. (C) Interaction fusion module: integrates the extracted drug and target features from (B) and simulates their interactions using BAN.

To mitigate the current limitations, we have developed PocketDTA, an advanced DTA prediction model. Specifically, (i) to enhance the generalization ability of the model, PocketDTA encodes the sequence information by drug Morgan fingerprints (Rogers and Hahn 2010) and through protein pre-trained model ESM-2 (Lin *et al.* 2023). As the pre-trained model learns in an unsupervised manner from millions of protein sequences, it generates rich sequence representations beyond the DTA dataset. (ii) To address the problem of insufficient 3D information, PocketDTA first applies the contrastive learning pre-trained framework GraphMVP (Liu *et al.* 2021) to generate rich and discriminative 3D structural representations of drugs. Further, a specific module GraphMVP-Decoder is introduced to extract drug 3D structure information. Second, we used the prediction tool DoGSite3 (Graef *et al.* 2023) to obtain the 3D structures of the top-3 target binding pockets. A customized geometric deep learning framework GVP-GNN Layers, is then used to learn the 3D structural information of these pockets. (iii) To address the issue of limited interpretability, this study combined global information (sequence) with local information (structure) to create a richer multimodal representation while using fewer model parameters. A bilinear attention network (BAN) (Kim *et al.* 2018) is introduced to model the local interactions between the drug and target, with the bilinear interaction weight matrix providing an interpretable analysis of these interactions. The overall flow of PocketDTA is illustrated in Fig. 1.

In this study, we performed refined preprocessing to the Davis and KIBA datasets, including operations such as noise data removal and protein structural domain extraction, to improve their quality. Using these datasets, we identified the optimal pre-trained models and the ideal number of pockets through a series of comparative experiments. In addition, recognizing that the uncertainty of the AlphaFold2 prediction model could impact results, this study assessed the effect of protein structures with varying confidence levels on model performance. Subsequently, the performance of the PocketDTA model was assessed, and the results indicated that PocketDTA outperformed SOTA methods across several evaluation metrics. Feature ablation and module ablation experiments further confirmed the effectiveness of the PocketDTA components. Even in more stringent cold-start scenarios, PocketDTA continued to show robust performance. Finally, the substantial interpretative potential of PocketDTA is validated through visual analyses of molecular docking and bilinear interaction weight matrices.

## 2 Materials and methods

### 2.1 Datasets

In this study, we used two publicly available benchmark datasets, Davis (Davis *et al.* 2011) and KIBA (Tang *et al.* 2014), to evaluate the performance of PocketDTA. These two datasets provide drug SMILES strings and target sequences. Data preprocessing was performed on both datasets, primarily comprising the following steps: (i) updating target sequences and removing redundant samples; (ii) using Alphafold2 to obtain the 3D coordinates of the target; (iii) extracting the protein domain portion of the target to obtain higher confidence 3D coordinates of the target; (iv) standardizing drug and target sequence lengths. Detailed descriptions are in Supplementary Material S1.1–S1.2, Supplementary Fig. S1, and Supplementary Tables S1 and S2.

### 2.2 Input representation

#### 2.2.1 Drug sequence representation

Drug sequence representation can be represented by molecular Morgan fingerprint (Rogers and Hahn 2010), which serves as an information-intensive tool for representation the molecular of compounds. It generates a vector where each position is a binary number (0 or 1), indicating the presence or absence of specific molecular substructures. The embedding for drug sequence representation is defined as $D_{\text{seq}}\in {\mathbb{R}}^{d_{\text{seq}}}$.

#### 2.2.2 Drug structure representation

In traditional drug structure representation methods, the focus is primarily on the 2D structure of the molecule, often overlooking the influence of the spatial structure of the molecule on its chemical properties. Conversely, the GraphMVP (Liu *et al.* 2021) pre-trained framework seeks to offer a richer and more accurate representation of the molecule by incorporating the 3D geometric information of the molecule. This pre-trained model captures the spatial relationships between atoms within a molecule and the collective impact of these relationships on the overall properties of the molecule.

We utilized RDKit (Landrum 2013) to transform SMILES strings into 2D molecular graphs *G*(***X***,***E)***, where the matrix ***X*** signifies the atom attributes, and matrix ***E*** represents the bond attributes. By retaining its pretrained parameters, the model accepts 2D molecular graphs as input and produces drug structure embeddings $D_{\text{stru}}\in R^{m\times d_{\text{stru}}}$, which include 3D conformations (m is the total number of drug heavy atoms).

#### 2.2.3 Target sequence representation

Currently, protein pre-trained models show remarkable advantages in computational biology, learning deep protein features from large-scale sequence data. Their strength lies in capturing complex protein patterns and enhancing downstream task accuracy and efficiency. We used ESM-2 (1280) (Lin *et al.* 2023) models to characterize target sequence embeddings $T_{\text{seq}}\in R^{p_{s}{\times t}_{\text{seq}}}$ (*p_s_* represents the target sequence length). To capture global protein sequence information and improve training efficiency, average pooling was applied along the protein length to obtain the embedding $T_{\text{seq}}\in R^{t_{\text{seq}}}$.

#### 2.2.4 Target structure representation

At present, although we have obtained high-quality 3D coordinate information of targets, effectively utilizing their structural information remains a critical challenge in DTA research. To efficiently utilize the target 3D structure information, the following steps are taken: (i) obtaining the 3D coordinates of the target binding pocket, (ii) encoding these coordinates as input into scalar and vector feature tuples (*s*, ***V***). Detailed operation of the above steps is shown in Supplementary Material S1.3.

### 2.3 Model architecture

**Figure 2.**
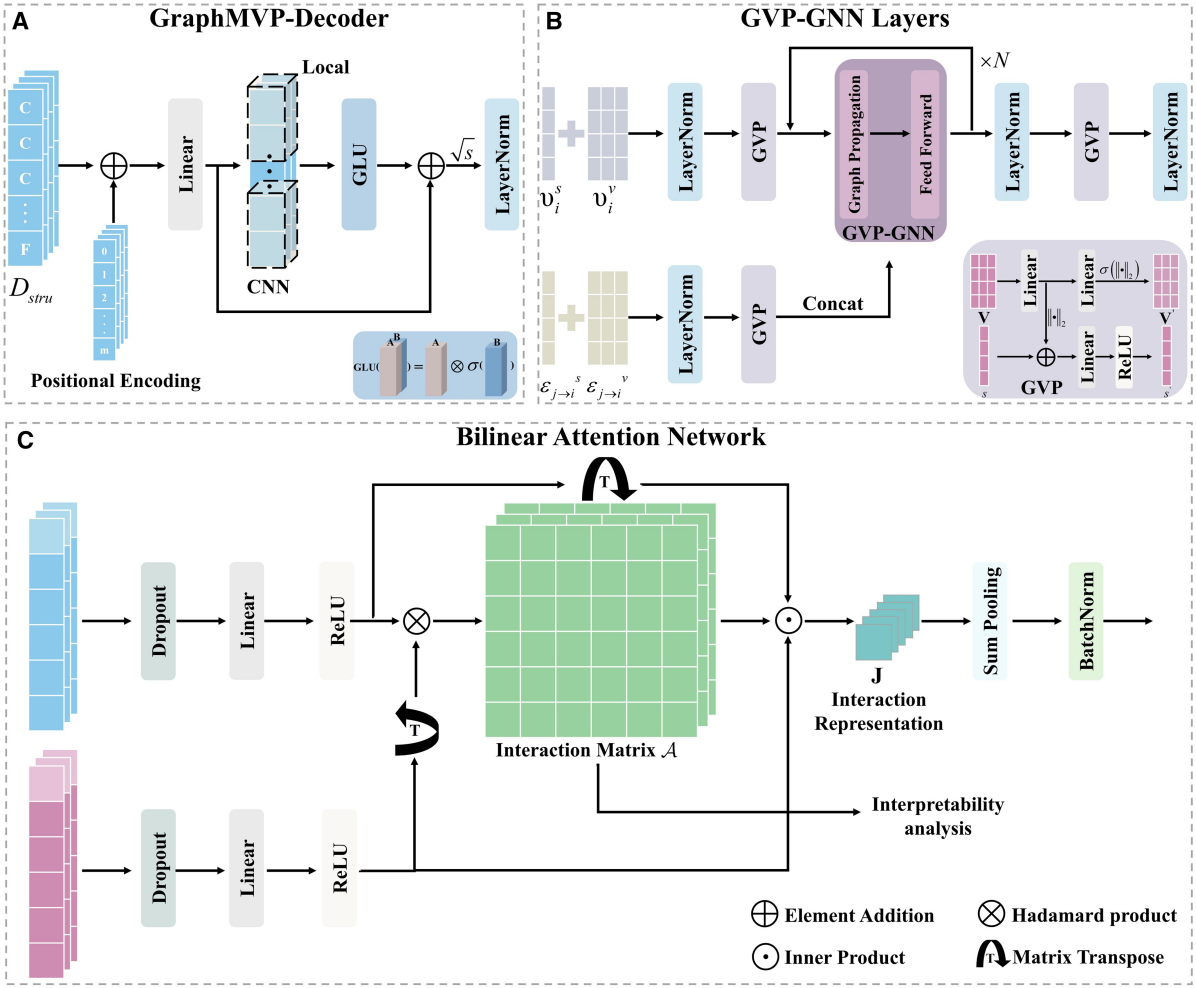
Framework of the main components (A) GraphMVP-Decoder: precisely captures subtle features of molecular 3D structures for accurate molecular representation. (B) GVP-GNN layers: utilizes geometric deep learning to extract detailed 3D information from the target pocket, enhancing the understanding of target structure. (C) Bilinear attention network: models local interactions between the drug and target.

In this section, we detail the feature extraction (Fig. 1B) and the interaction fusion (Fig. 1C), focusing primarily on three innovative components: the GraphMVP-Decoder (Fig. 2A), GVP-GNN Layers (Fig. 2B), and BAN (Fig. 2C).

For the drug sequence representation *D*_seq_ and target sequence representation *T*_seq_ obtained above, owing to the rich representations facilitated by transfer learning, only a simple fully connected layer is necessitated. Two fully connected layers along with a ReLU activation function are used to transform them into $D_{s\text{eq}}^{*}$$\in R^{h_{d}}$ and $T_{s\text{eq}}^{*}$$\in R^{h_{t}}$, as shown in Equations (1) and (2),

(1) $$D_{s\text{eq}}^{*}\in W_{d_{2}}(\text{ReLU}(W_{d_{1}}D_{\text{seq}}+b_{d_{1}}))+b_{d_{2}},$$

(2) $$T_{s\text{eq}}^{*}\in W_{t_{2}}(\text{ReLU}(W_{t_{1}}T_{\text{seq}}+b_{t_{1}}))+b_{t_{2}},$$

where weight matrices $W_{d_{1}}\in R^{512\times d_{\text{seq}}}$, $W_{d_{2}}\in R^{h_{d}\times 512}$, $W_{t_{2}}\in R^{256\times t_{\text{seq}}}$, $W_{t_{2}}\in R^{h_{t}\times 256}$, and bias vectors $b_{d_{1}}\in R^{512}$, $b_{d_{2}}\in R^{h_{d}}$, $b_{t_{1}}\in R^{256}$, $b_{t_{2}}\in R^{h_{t}}$.

#### 2.3.1 GraphMVP-decoder

To precisely capture the subtle features of molecular 3D structures, we developed the GraphMVP-Decoder. The decoder initially computes the positional embedding $\mathrm{Po}s_{\mathrm{emb}}$ from the drug structure representation $D_{\mathrm{stru}}$ and then integrates it to preserve spatial location information. Following this, the decoder uses a fully connected layer to reduce dimension and carry out a preliminary extraction of local features:

(3) $$D_{\text{stru}}^{\text{Pos}}=W_{d_{3}}\left(D_{\text{stru}}+P{\mathrm{os}}_{\text{emb}}\right)+b_{d_{3}},$$

where weight matrix $W_{d_{3}}\in R^{h_{d}\times d_{\text{stru}}}$, bias vector $b_{d_{3}}\in R^{h_{d}}$. The CNN sliding window mechanism is used for further refinement of the drug local features. In addition, the GLU (Dauphin *et al.* 2016) activation function is used to accommodate the data complexity, with its gating mechanism effectively filtering the input and highlighting key information compared to other activation functions. At the same time, residual connectivity, scaling factor, and LayerNorm are introduced to improve the stability of training and accelerate the convergence speed. The decoder can be formulated as

(4) $$D_{\text{stru}}^{*}=\text{LayerNorm}\left[\text{GLU}\left(\text{CNN}\left(D_{\text{stru}}^{\text{Pos}}\right)\right)\right]+D_{\text{stru}}^{\text{Pos}}\cdot \sqrt{s},$$

where *s* = 1/2 is the scaling factor, GLU(A,B)=A⊗σ(B),σ(⋅), σ(·) is Sigmoid function, $D_{\text{stru}}^{*}\in R^{m\times h_{d}}$.

#### 2.3.2 GVP-GNN layers

In order to thoroughly investigate the geometric and spatial features of the target pockets, an advanced Geometric Vector Perceptron (GVP) (Jing *et al.* 2020) is utilized to model the protein graph G=(V,E). The GVP is considered an extension of the linear transformation, processing feature tuples $(s,V)\in R^{\eta }\times R^{\mu \times 3}$ into new tuples $(s^{\prime },V^{\prime })\in R^{\eta^{\prime }}\times R^{\mu^{\prime }\times 3}$. Detailed calculations are shown in the Supplementary Material S1.4. Most critically, GVP possesses a desirable property: its scalar and vector outputs demonstrate invariance and equivariance with respect to rotation and reflection operations in 3D Euclidean space. Specifically, if $\mathrm{GVP}(s,V)=(s^{ʹ},V^{ʹ})$, then for any rotation and reflection transformation R, it holds that $\mathrm{GVP}(s,R(V))=(s^{ʹ},{R(V)}^{ʹ})$ (Jing *et al.* 2020). Invariance indicates that the scalar *s* remains constant under transformation R, meaning the output scalar is unaffected by such transformations. Equivariance ensures that any transformation R applied to the vector V results in corresponding transformation in the output vectors. This property endows GVP with greater expressiveness in learning geometric features in 3D space compared to other neural network models.

GVP-GNN uses GVP to enhance the model recognition of geometric structural features, utilizing a message-passing mechanism to update node embeddings. This GVP-GNN architecture comprises two layers: a graph propagation layer and a feedforward layer. The graph propagation layer initially computes a message derived from the node and edge embeddings, then applies this message to update the node embeddings. Specifically, the graph propagation layer of layer l can be represented as

(5) $$m_{j\to i}^{\left(l\right)}=g\left(\mathrm{Concat}\left(\upsilon_{j}^{\left(l-1\right)},\varepsilon_{j\to i}\right)\right),$$

(6) $$\upsilon_{i}^{\left(l\right)}=\mathrm{LayerNorm}\left(\upsilon_{i}^{\left(l-1\right)}+\frac{1}{\left|N\left(i\right)\right|}\sum_{j\in N\left(i\right)}m_{j\to i}^{\left(l\right)}\right),$$

where $\upsilon_{j}^{\left(l-1\right)}$ is the embedding of node j at layer l−1, $\varepsilon_{j\to i}$ represents the edge embedding from the node j to node i, $g\left(\cdot \right)$ denotes three stacked GVP layers, $m_{j\to i}^{\left(l\right)}$ symbolizes the message conveyed from node j to node i at layer l, and $N\left(i\right)$ signifies the set of neighboring nodes of the node i. Subsequently, the feedforward layer proceeds to refine the node embeddings, which can be delineated as

(7) $$\upsilon_{i}^{\left(l\right)}=\mathrm{LayerNorm}\left(\upsilon_{i}^{\left(l\right)}+\mathrm{Dropout}\left(g\left(\upsilon_{i}^{\left(l\right)}\right)\right)\right),$$

where $g\left(\cdot \right)$ denotes two stacked GVP layers.

We developed customized GVP-GNN Layers using GVP and GVP-GNN, utilizing the protein graph $G=\left(V,E\right)$ as input to learn the 3D structural information of the target binding pockets. Initially, preliminary extraction of edge and node feature embeddings was conducted through a single GVP layer. Subsequently, this data is co-inputted into the N-layer GVP-GNN for information fusion and updating of node and edge embeddings. Finally, the integrated information is further processed through the GVP layer, and the framework can be depicted as

(8) $$E^{\prime }=\mathrm{GVP}\left(\mathrm{LayerNorm}\left(E\right)\right),$$

(9) $$V^{\prime }=\mathrm{GVP}\left(\mathrm{LayerNorm}\left(V\right)\right),$$

(10) $$T_{\mathrm{stru}}=\mathrm{LayerNorm}\left(\mathrm{GVP}\left(\mathrm{LayerNorm}\left(\mathrm{GVPGNN}\left(V^{\prime },E^{\prime }\right)\right)\right)\right),$$

where $\mathrm{GVPGNN}\left(\cdot \right)$ is the N-layer GVP-GNN, $\mathrm{GVP}\left(\cdot \right)$ of Equation (10) only generates scalar features, $T_{\mathrm{stru}}\in R^{p\times h_{s}}$ (p represents the target binding pockets sequence length).

To facilitate subsequent feature fusion, the same operation as used in target sequence representation is used to transform ${T_{\mathrm{stru}}}^{*}\in R^{p\times h_{t}}$,

(11) $${T_{\mathrm{stru}}}^{*}=W_{t_{4}}\left(\text{ReLU}\left(W_{t_{3}}T_{\mathrm{stru}}+b_{t_{3}}\right)\right)+b_{t_{4}},$$

where weight matrices $W_{t_{3}}\in R^{128\times h_{s}}$, $W_{t_{4}}\in R^{h_{t}\times 128}$, and bias vectors $b_{t_{3}}\in R^{128}$, $b_{t_{4}}\in R^{h_{t}}$.

#### 2.3.3 Bilinear attention network

To construct multimodal representations of drug targets containing global and local information, the following operations are performed:

(12) $$D=\mathrm{Concat}\left({D_{\mathrm{seq}}}^{*},{D_{\mathrm{stru}}}^{*}\right),$$

(13) $$T=\mathrm{Concat}\left({T_{\mathrm{seq}}}^{*},{T_{\mathrm{stru}}}^{*}\right),$$

where $D\in R^{\left(m+1\right)\times h_{d}}$, $T\in R^{\left(p+1\right)\times h_{t}}$.

The BAN, originally designed for visual question answering, was used to capture local interactions between drugs and targets (Bai *et al.* 2023). It captures rich interaction information in multichannel data while maintaining the computational efficiency of a single attention mechanism. It consists of two main parts: (i) pairwise interaction matrix A acquisition, (ii) interaction representation J extraction. Inputs $D={\left(d_{1},d_{2},\ldots ,d_{m+1}\right)}^{Τ}$ and $T={\left(t_{1},t_{2},\ldots ,t_{p+1}\right)}^{Τ}$ are each mapped to a shared feature space using simple linear layers and ReLU activation functions. We then compute the pairwise interaction matrix $A\in R^{\left(m+1\right)\times \left(p+1\right)}$ between them using the Hadamard product,

(14) $$A=\left[\left(1\cdot q^{Τ}\right)⊗\left(\text{ReLU}\left(W_{d_{3}}\cdot Dropout\left(D\right)\right)\right)\right]\cdot {\left(\text{ReLU}\left(W_{t_{5}}D\mathrm{ropout}\left(T\right)\right)\right)}^{Τ},$$

where $1\in R^{\left(m+1\right)\times 1}$ is an all-ones vector, $q\in R^{1\times K}$ represents the learnable weight vector, ⊗ denotes Hadamard product, weight matrices $W_{d_{3}}\in R^{K\times h_{d}}$, $W_{t_{5}}\in R^{K\times h_{t}}$ and $\mathrm{Dropout}\left(\cdot \right)$ is a stochastic discard operation. To facilitate a clearer understanding of the bilinear interaction weight matrix, the elements in A can be expressed as:

(15) $$A_{i,j}=q^{Τ}\cdot \left[\left(\text{ReLU}\left(W_{d_{3}}\cdot \mathrm{Dropout}\left(d_{i}\right)\right)\right)⊗{\left(\text{ReLU}\left(W_{t_{5}}\cdot \mathrm{Dropout}\left(t_{j}\right)\right)\right)}^{Τ}\right],$$

the element $A_{i,j}$ in matrix A describes the interaction strength between drug heavy atom i and target amino acid residue j. This specifically reflecting the interaction between drug heavy atoms and amino acid residues. Consequently, the pairwise interaction matrix A facilitates interpretable analysis of the DTI mechanism.

Unlike the conventional attention mechanism, which involves multiplying the weight matrix A by itself to obtain a new representation, BAN constructs the interaction representation $J={\left(j_{1},j_{2},\ldots ,j_{K}\right)}^{Τ}\in {\mathbb{R}}^{K}$ through a bilinear product:

(16) $$j_{k}={{\left(\text{ReLU}\left(W_{d_{3}}\cdot Dropout\left(D\right)\right)\right)}^{Τ}}_{k}\;\cdot A\;\cdot {\left(\text{ReLU}\left(W_{t_{5}}\cdot \mathrm{Dropout}\left(T\right)\right)\right)}_{k},$$

where${{\left(\text{ReLU}\left(W_{d_{3}}\cdot \mathrm{Dropout}\left(D\right)\right)\right)}_{k}}^{Τ}$ is the kth row of ${\left(\text{ReLU}\left(W_{d_{3}}\cdot \mathrm{Dropout}\left(D\right)\right)\right)}^{Τ}$, ${\left(\text{ReLU}\left(W_{t_{5}}\cdot \mathrm{Dropout}\left(T\right)\right)\right)}_{k}$ is the kth column of $\left(\text{ReLU}\left(W_{t_{5}}\cdot \mathrm{Dropout}\left(T\right)\right)\right)$. This step introduces no new learning parameters, as the weights are shared with those from the previous step to reduce the model parameter.

Subsequently, pooling and normalization operations are conducted on the interaction representation vector J to yield $J^{\prime }$:

(17) $$J^{\prime }=\mathrm{BatchNorm}\left(\mathrm{SumPool}\left(J,s\right)\right),$$

where the $\mathrm{SumPool}\left(\cdot \right)$ is a sum pooling with stride s=3, $\mathrm{BatchNorm}\left(\cdot \right)$ is batch normalization, $J^{\prime }\in R^{K/s}$ ($K/s=h_{b}$ is the BAN output dimension). In this paper, we used the multi-head form. Since the weight matrices $W_{d_{3}}$ and $W_{t_{5}}$ are shared, each additional head only requires a new weight vector q, reducing the number of model parameters.

Finally, the interaction representations $J^{\prime }$ are directly inputted to the MLP to generate predicted values y,

(18) $$y=\mathrm{MLP}\left(J^{\prime }\right),$$

where the MLP comprises three layers stacked sequentially: a fully connected layer, a ReLU activation function, a Dropout layer, and another fully connected layer. The dimensions of the four fully connected layers are 1024, 1024, 256, and 1, respectively.

### 2.4 Evaluation metrics

In our study, to comprehensively assess the performance of the prediction model, we used statistical measures such as mean square error (MSE), consistency index (CI), $r_{m}^{2}$, Pearson correlation coefficient (Pearson), and Spearman rank correlation coefficient (Spearman). They are described in detail in Supplementary Material S1.5.

### 2.5 Experiment setting

In previous DTA studies, the benchmark datasets were usually divided into training and test sets. To more accurately reflect the model real performance and improve experimental robustness, this study split the benchmark datasets into training, validation, and test sets at an 8:1:1 ratio. All experiments were conducted five times with different random seeds. The mean and variance of these results were used as the model final evaluation metrics, indicating its accuracy and stability, respectively (Xu and Goodacre 2018, He *et al.* 2023). During the model training phase, MSE was used as the loss function over 200 epochs. RAdam (Liu *et al.* 2019) ($\beta_{1}=0.9$, $\beta_{2}=0.999$, $\varepsilon =10^{-8}$) served as the base optimizer, enhanced by the advanced optimization strategy Lookahead (Zhang *et al.* 2019) (k=5, α=0.5), to improve stability during training. All hyperparameters were optimized using the grid search method, with the search range and selected values detailed in Supplementary Table S3. Implemented in Python 3.7.12 and PyTorch 1.13.1 (Paszke *et al.* 2019), the proposed model was trained on a Linux system with an NVIDIA A800-80G PCIE GPU and an Intel_5318Y 2.1 GHz CPU.

## 3 Results

### 3.1 Comparison experiment

#### 3.1.1 Comparative analysis of various representation methods

**Figure 3.**
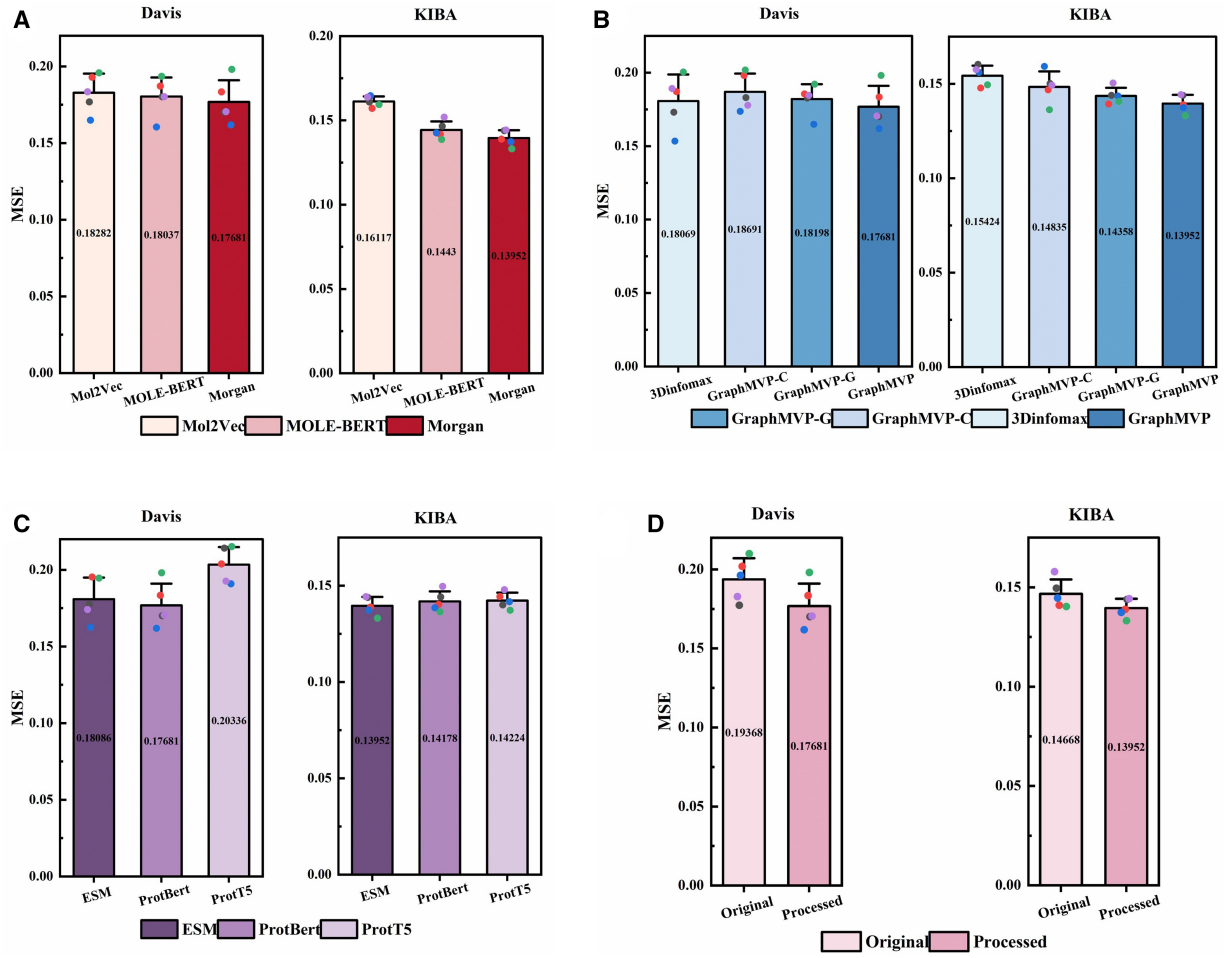
Comparative analysis of MSE bar-box plots for various representation methods on benchmark datasets. Five of the dots represent results from different random seeds. The more concentrated the data points and the shorter the bar, the better the performance is represented. (A) Drug sequence representation. (B) Drug structure representation. (C) Target sequence representation. (D) Target structure representation.

We investigated the sequence and structural features of various drugs and their targets to identify the features most appropriate for the DTA task. For drug sequence representation, we evaluated Morgan, Mol2Vec (Jaeger *et al.* 2018), and Mole-BERT (Xia *et al.* 2023) molecular embeddings. The findings reveal that Morgan fingerprints outperformed the other methods across all performance evaluation metrics on the two benchmark datasets (Fig. 3A illustrates a comparison of the MSE results). This observation suggests that, despite the considerable complexity of many molecular pre-trained models, their representational capacity in the DTA domain may be lacking when compared to the conventional Morgan fingerprint.

For the drug structure representation, we compared GraphMVP, GraphMVP-C (Liu *et al.* 2021), GraphMVP-G (Liu *et al.* 2021), and 3Dinfomax (Strk *et al.* 2021) approaches. GraphMVP-C and GraphMVP-G are variants of GraphMVP that incorporate a 2D graph training strategy. Among these, GraphMVP showed superior performance, surpassing the other techniques (Fig. 3B). This result may be attributed to the similarity of the 2D topology of the benchmark dataset, and the additional 2D topological information rather increases the noise of the model.

For the target sequence representation, we evaluated the embeddings of the leading protein pre-trained models: ESM-2, ProtBert (1024) (Elnaggar *et al.* 2021), and ProtT5 (1024) (Elnaggar *et al.* 2021). Figure 3C shows that ProtBert outperformed the other models on the Davis dataset (MSE of 0.177), while ESM-2 performed best on the KIBA dataset (MSE of 0.140). ProtBert and ESM-2 were selected as the methods for protein sequence representation for the Davis and KIBA datasets, respectively. Furthermore, minimal performance differences were observed among these extensive protein pre-trained models on the data-rich KIBA dataset. This further substantiates the notion that pre-trained models offer a more robust representation for downstream tasks in contexts where data is plentiful.

**Figure 4.**
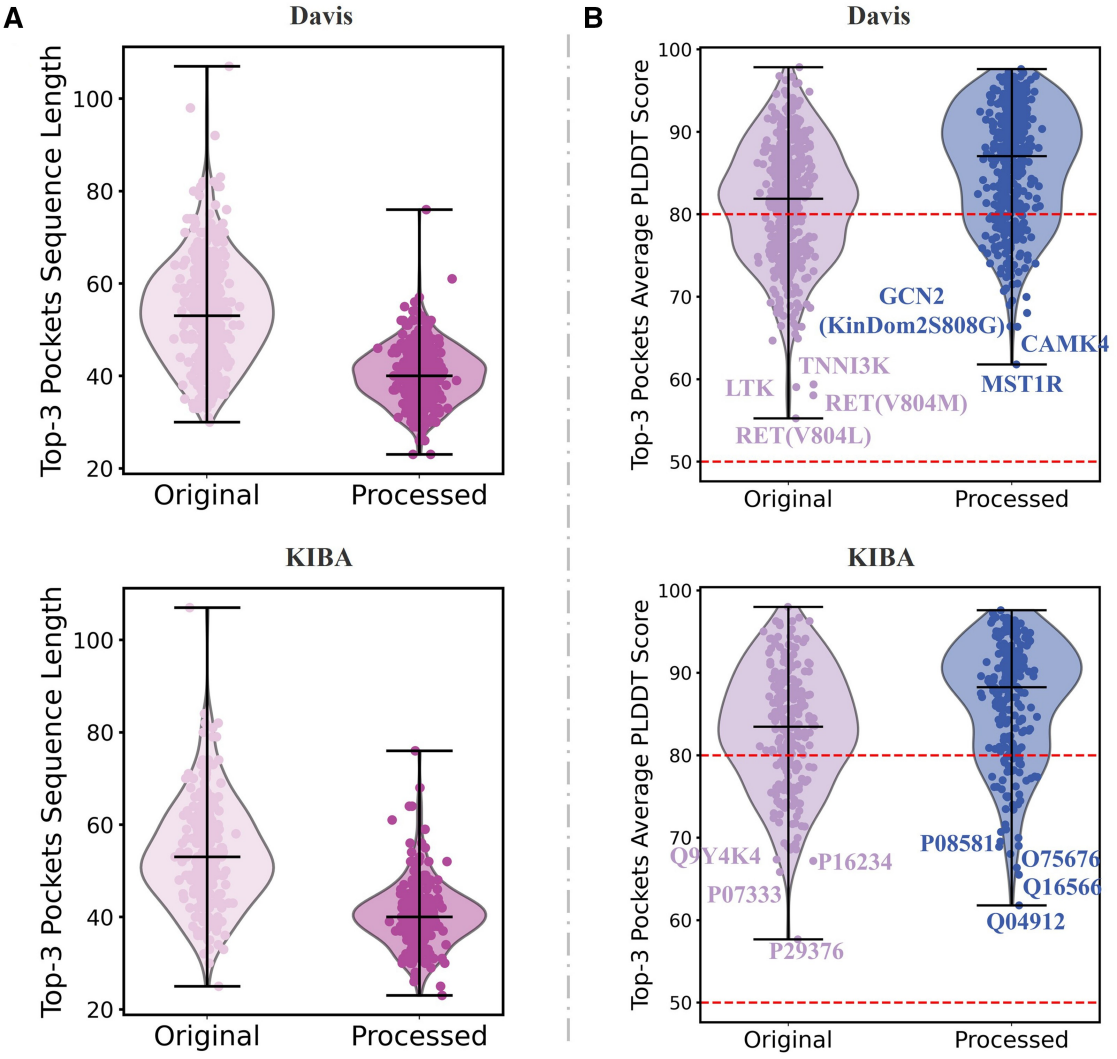
Comparison of the distributions of predicted binding pocket sequence lengths and average pLDDT values as depicted by violin plots, obtained from protein structures with high or low confidence. (A) Binding pocket sequence lengths on benchmark datasets. The processed protein pocket sequences are notably shorter and exhibit a more concentrated length distribution. (B) Average pLDDT values on benchmark datasets. The processed protein pockets display higher average pLDDT values, indicating improved structural confidence.

Lastly, for target structure representation, in Supplementary Material S1.2, we obtained higher-confidence protein 3D coordinates by extracting protein structural domains. We would like to conduct a comparative analysis of the impact of unprocessed lower-confidence protein 3D coordinates on the experimental results. Figure 4 shows the total sequence lengths and the distributions of average pLDDT values of top-3 binding pockets, derived from original and processed protein 3D coordinate predictions. Further analysis indicates that the pockets obtained from the processed protein have shorter lengths and higher average pLDDT values (primarily concentrated around 90). The comparative analysis of model performance, using input from pockets information at two distinct confidence levels, is presented in Fig. 3D. The outcomes suggest that structural information at a higher confidence level can enhance performance, indicating that improvements in data quality are crucial for advancing the DTA domain. The detailed results of this section are available in Supplementary Tables S4–S7.

#### 3.1.2 Evaluating the impact of predicted pocket quantity variations

As shown in Table 1, using top-3 predicted binding pockets as target structure inputs resulted in the best performance across all evaluation metrics. As the number of pockets decreases, performance deteriorates, with the results for top2 being slightly worse than top-3. Considering that increasing the number of pockets leads to a surge in the amount of data, it defeats the purpose for which we designed the model. Furthermore, since top-3 includes most binding sites, the decision was made to select the top-3 binding pockets. In the future, advancements in computational resources are expected to facilitate the integration of more pockets into the model.

**Table 1.** Comparative analysis of predictive outcomes across varied pocket quantity scenarios on benchmark datasets.

Dataset	Pocket quantity	MSE↓	CI↑	$r_{m}^{2}$ ↑	Pearson↑	Spearman↑
Davis	Top-1	0.183 ± 0.012	0.902 ± 0.004	0.718 ± 0.023	0.883 ± 0.008	0.706 ± 0.006
	Top-2	0.182 ± 0.013	0.901 ± 0.004	0.713 ± 0.024	0.885 ± 0.006	0.705 ± 0.008
	Top-3	**0.177** ± **0.013**	**0.903** ± **0.005**	**0.731** ± **0.017**	**0.887** ± **0.008**	**0.708** ± **0.005**
KIBA	Top-1	0.148 ± 0.006	0.890 ± 0.002	0.746 ± 0.016	0.890 ± 0.003	0.881 ± 0.003
	Top-2	0.142 ± 0.006	0.891 ± 0.001	0.768 ± 0.012	0.894 ± 0.003	0.883 ± 0.002
	Top-3	**0.140** ± **0.004**	**0.892** ± **0.002**	**0.771** ± **0.011**	**0.896** ± **0.003**	**0.885** ± **0.003**

Bold corresponds to the best performance for each metric, and ↑/↓ indicates that the larger/smaller the metrics, the better the model performance.

#### 3.1.3 Comparative assessment of performance relative to SOTA methods

A comparative analysis was conducted against the SOTA methods: DeepDTA (Öztürk *et al.* 2018), GraphDTA (Nguyen *et al.* 2020), FusionDTA (Yuan *et al.* 2022), MGraphDTA (Yang *et al.* 2022), 3DProtDTA (Voitsitskyi *et al.* 2023), NHGNNDTA (He *et al.* 2023), and MDFDTA (Ranjan *et al.* 2024) using a consistent experimental setup across two benchmark datasets. Among these approaches, FusionDTA leverages pretrained protein representations, whereas MDFDTA integrates pretrained representations of both proteins and molecules, providing a more comprehensive basis for comparative experiments. Specifically, the comparative models were retrained following the parameters specified in existing literature, and the results are summarized in Table 2. In the evaluation of the Davis dataset, the PocketDTA model demonstrates improved performance in terms of MSE, CI, and Pearson but slightly underperforms compared to the NHGNNDTA and MDFDTA methods on the $r_{m}^{2}$and Spearman metrics. We note that compared to the next best-performing MDFDTA method, PocketDTA reduces MSE by only 0.562%, improves CI by only 0.111%, and Pearson by only 0.226%. Furthermore, PocketDTA demonstrates significant variations across all assessment metrics relative to DeepDTA and GraphDTA (Student’s *t*-test, *P* < 0.05), but it does not show significant differences in most metrics compared to FusionDTA, MGraphDTA, 3DProtDTA, MDFDTA, and NHGNNDTA (*P* > 0.05). This phenomenon is due to the unbalanced distribution of the Davis dataset and its limited sample size. This limits the upper-performance threshold of the models, making significant improvements in model performance difficult. However, compared to the SOTA model regarding the number of trainable parameters and training time (Supplementary Table S8), PocketDTA demonstrates an advantage in training efficiency, attributable to the simplicity of its model.

**Table 2.** Comparative performance analysis of PocketDTA and other SOTA models on the benchmark datasets.

Dataset	Methods	MSE↓	CI↑	$r_{m}^{2}$ ↑	Pearson↑	Spearman↑
Davis	DeepDTA	0.238 ± 0.026	0.880 ± 0.010	0.693 ± 0.033	0.843 ± 0.019	0.676 ± 0.016
	GraphDTA	0.240 ± 0.008	0.880 ± 0.006	0.654 ± 0.015	0.844 ± 0.006	0.675 ± 0.014
	FusionDTA	0.181 ± 0.013	0.901 ± 0.003	0.750 ± 0.013	0.880 ± 0.003	0.709 ± 0.015
	MGraphDTA	0.189 ± 0.009	0.896 ± 0.004	0.750 ± 0.012	0.878 ± 0.007	0.697 ± 0.006
	3DProtDTA	0.193 ± 0.016	0.900 ± 0.005	0.746 ± 0.021	0.877 ± 0.009	0.705 ± 0.007
	NHGNNDTA	0.179 ± 0.011	0.902 ± 0.003	0.759 ± 0.016	0.885 ± 0.005	**0.711** ± **0.014**
	MDFDTA	0.178 ± 0.012	0.902 ± 0.002	**0.760** ± **0.015**	0.884 ± 0.006	0.710 ± 0.007
	PocketDTA	**0.177** ± **0.013**	**0.903** ± **0.005**	0.731 ± 0.017	**0.887** ± **0.008**	0.708 ± 0.005
KIBA	DeepDTA	0.195 ± 0.004	0.850 ± 0.003	0.704 ± 0.010	0.851 ± 0.004	0.830 ± 0.006
	GraphDTA	0.179 ± 0.005	0.862 ± 0.004	0.731 ± 0.009	0.864 ± 0.005	0.847 ± 0.006
	FusionDTA	0.155 ± 0.006	0.880 ± 0.002	0.759 ± 0.016	0.889 ± 0.003	0.880 ± 0.003
	MGraphDTA	0.152 ± 0.005	0.882 ± 0.003	0.760 ± 0.006	0.886 ± 0.003	0.875 ± 0.004
	3DProtDTA	0.158 ± 0.005	0.880 ± 0.001	0.762 ± 0.012	0.882 ± 0.003	0.873 ± 0.002
	NHGNNDTA	0.157 ± 0.008	0.879 ± 0.003	0.753 ± 0.021	0.882 ± 0.007	0.870 ± 0.005
	MDFDTA	0.151 ± 0.009	0.889 ± 0.003	0.765 ± 0.019	0.884 ± 0.007	0.871 ± 0.006
	PocketDTA	**0.140** ± **0.004**	**0.892** ± **0.002**	**0.771** ± **0.011**	**0.896** ± **0.003**	**0.885** ± **0.003**

Bold corresponds to the best performance for each metric, and ↑/↓ indicates that the larger/smaller the metrics, the better the model performance.

**Figure 5.**
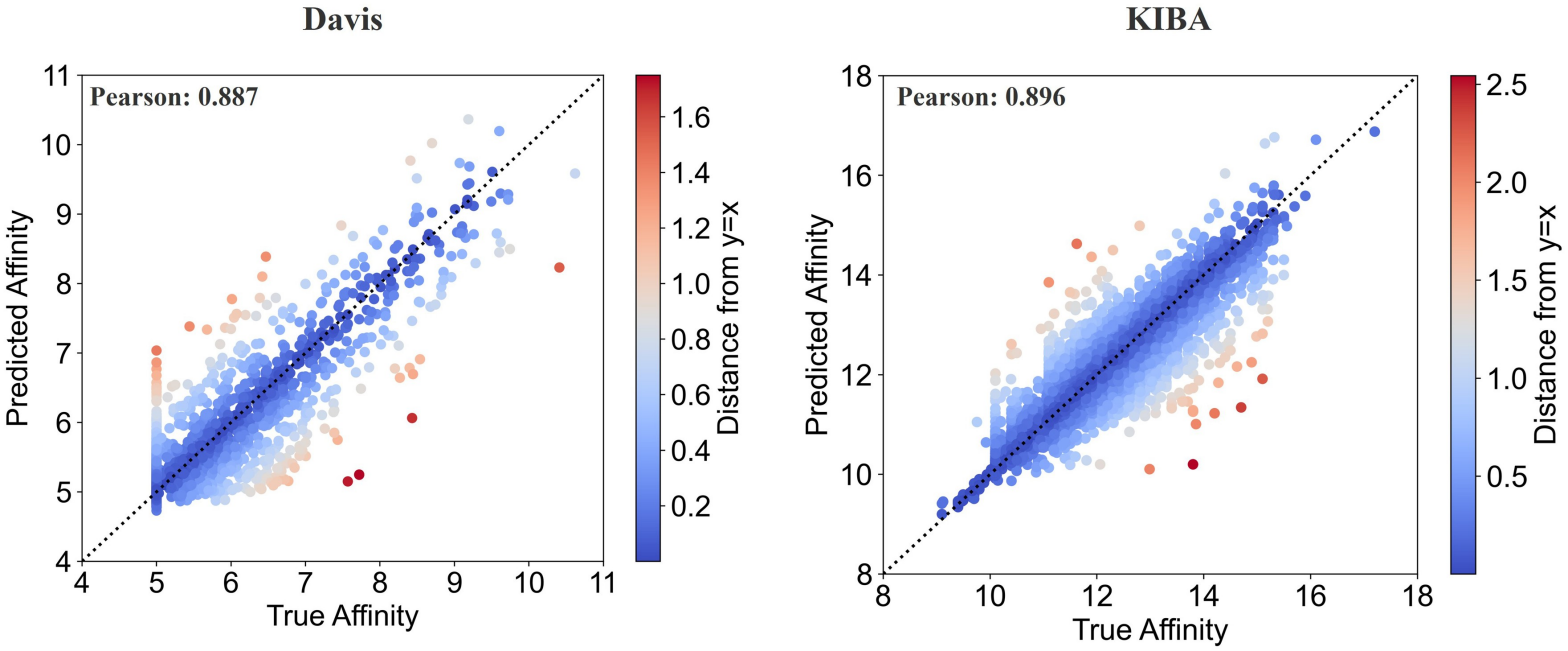
Scatterplot of PocketDTA predicted affinity versus true affinity on the benchmark datasets, where darker-red data points indicate larger prediction errors, and darker-blue data points indicate smaller prediction errors.

In the KIBA dataset, PocketDTA shows significant improvement in all evaluation metrics compared to the SOTA methods. Compared with the next best performing MDFDTA method, PocketDTA shows a 7.285% reduction in MSE, improvements of 0.337% in CI, 0.784% in ${r_{m}}^{2}$, and 0.787% and 0.568% in Pearson and Spearman, respectively. Meanwhile, the decrease in variance implies a higher stability of PocketDTA. It is noteworthy that PocketDTA is statistically significantly superior (*P* < 0.05) to the SOTA method in terms of MSE, CI, Pearson, and Spearman metrics. Figure 5 presents the scatter plot comparing the predicted and actual values of PocketDTA on the test set.

In addition, to demonstrate the effectiveness of this preprocessing measure (Supplementary Material S1.2), we compared the performance changes between the original and preprocessed benchmark datasets using SOTA models. The detailed analyses are presented in Supplementary Material S2.1, Supplementary Fig. S2, and Supplementary Table S9.

### 3.2 Ablation study

#### 3.2.1 Representation ablation study

To evaluate the influence of sequence and structural features on PocketDTA predictive accuracy, we performed feature ablation studies. The results unequivocally demonstrate that each feature is indispensable in the PocketDTA model. Detailed analyses are shown in Supplementary Material S2.2 and Supplementary Table S10.

#### 3.2.2 Module ablation study

**Figure 6.**
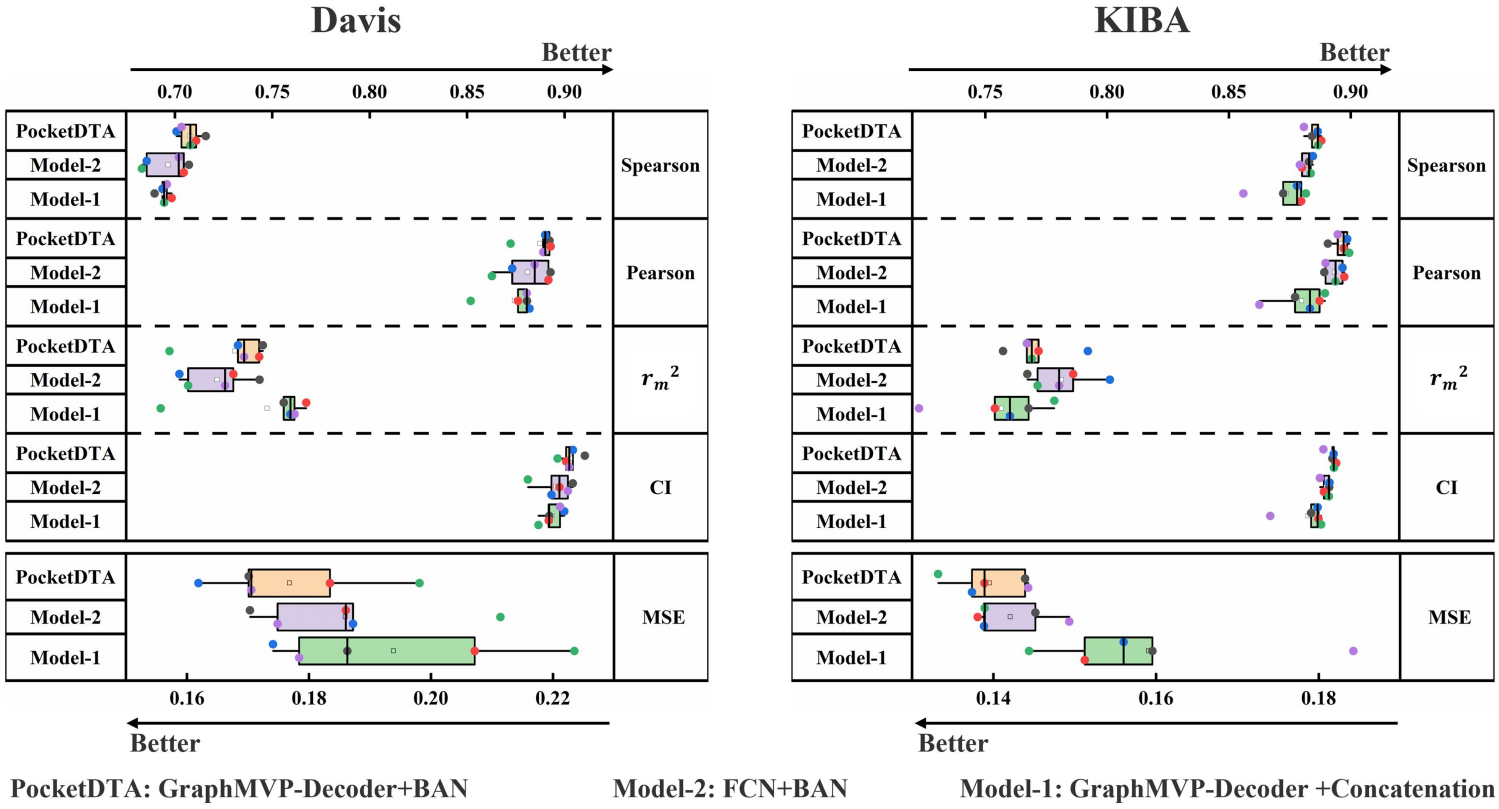
These charts present a comparative analysis of PocketDTA, Model-1, and Model-2 across various evaluation metrics, effectively demonstrating the contribution of each module within PocketDTA.

Module ablation studies were conducted to explore the impact of the proposed innovative module on the performance of the PocketDTA model. Specifically, the variant Model-1 uses a concatenation strategy for the interaction representation of drugs and targets. The Model-2 was proposed to replace the FCN module with GraphMVP-Decoder. The boxplots (Fig. 6) show that the PocketDTA model exhibits higher prediction accuracy and stability than Model-1 and Model-2, which validate the effectiveness of BAN and GraphMVP-Decoder. Detailed results can be found in Supplementary Table S11. For the GVP-GNN layers, the effectiveness of the GVP-GNN layers has been validated in the feature ablation section.

### 3.3 Cold start experiment

In practical applications of drug screening, a large number of drugs or targets may not be included in the training set. Therefore, a challenge for DTA models is to assess whether the excellent performance of the model on a specific dataset can generalize to unseen data. To this end, three novel dataset segmentation strategies were used: Cold drug, cold target, and all cold, to evaluate the generalization performance of DTA models. Detailed cold start setup is shown in the Supplementary Material S2.3. Table 3 presents cold-start experiment results on the KIBA dataset. Compared to other benchmark models, PocketDTA exhibits superior prediction performance under all three data segmentation methods, highlighting the model robustness in adapting to new environments. Specifically, the MSE of the PocketDTA model decreased by 4.857% (from 0.350 to 0.333) compared to the suboptimal model when addressing unknown drugs. When addressing unknown targets, the MSE decreased by 2.965% (from 0.371 to 0.360). Specifically, for unknown drug–target pairs, the MSE of PocketDTA decreased by 10.440% (from 0.546 to 0.489) compared to the suboptimal model. Similar prediction results were obtained on the Davis dataset (Supplementary Table S12). These results confirm that PocketDTA has stronger generalization capabilities than other models by leveraging pre-trained drug and target representations and integrating multimodal drug target features.

**Table 3.** Performance evaluation on more realistic settings of KIBA datasets.

Setting	Methods	MSE↓	CI↑	$r_{m}^{2}$ ↑	Pearson↑	Spearman↑
Cold drug	DeepDTA	0.416 ± 0.025	0.751 ± 0.006	0.390 ± 0.028	0.652 ± 0.021	0.635 ± 0.012
	GraphDTA	0.374 ± 0.013	0.773 ± 0.005	0.451 ± 0.034	0.692 ± 0.016	0.680 ± 0.010
	3DProtDTA	0.350 ± 0.009	0.780 ± 0.009	0.476 ± 0.034	0.726 ± 0.016	0.690 ± 0.020
	NHGNNDTA	0.385 ± 0.020	0.766 ± 0.011	0.433 ± 0.024	0.686 ± 0.016	0.664 ± 0.022
	PocketDTA	**0.333** ± **0.006**	**0.789** ± **0.008**	**0.495** ± **0.021**	**0.733** ± **0.008**	**0.711** ± **0.017**
Cold target	DeepDTA	0.417 ± 0.046	0.701 ± 0.018	0.371 ± 0.044	0.633 ± 0.027	0.521 ± 0.047
	GraphDTA	0.518 ± 0.075	0.646 ± 0.026	0.252 ± 0.033	0.513 ± 0.035	0.387 ± 0.064
	3DProtDTA	0.371 ± 0.072	0.734 ± 0.013	0.445 ± 0.060	0.692 ± 0.035	0.593 ± 0.033
	NHGNNDTA	0.386 ± 0.068	0.724 ± 0.014	0.422 ± 0.024	0.668 ± 0.028	0.570 ± 0.028
	PocketDTA	**0.360** ± **0.086**	**0.754** ± **0.017**	**0.454** ± **0.075**	**0.699** ± **0.042**	**0.635** ± **0.040**
All cold	DeepDTA	0.603 ± 0.127	0.621 ± 0.039	0.164 ± 0.087	0.406 ± 0.121	0.325 ± 0.103
	GraphDTA	0.667 ± 0.129	0.587 ± 0.011	0.104 ± 0.038	0.322 ± 0.067	0.236 ± 0.026
	3DProtDTA	0.546 ± 0.121	0.661 ± 0.019	0.243 ± 0.057	0.517 ± 0.059	0.424 ± 0.046
	NHGNNDTA	0.572 ± 0.114	0.653 ± 0.020	0.211 ± 0.054	0.471 ± 0.068	0.405 ± 0.047
	PocketDTA	**0.489** ± **0.099**	**0.680** ± **0.018**	**0.293** ± **0.067**	**0.556** ± **0.052**	**0.474** ± **0.042**

Bold corresponds to the best performance for each metric, and ↑/↓ indicates that the larger/smaller the metrics, the better the model performance.

## 4 Interpretability analysis

**Figure 7.**
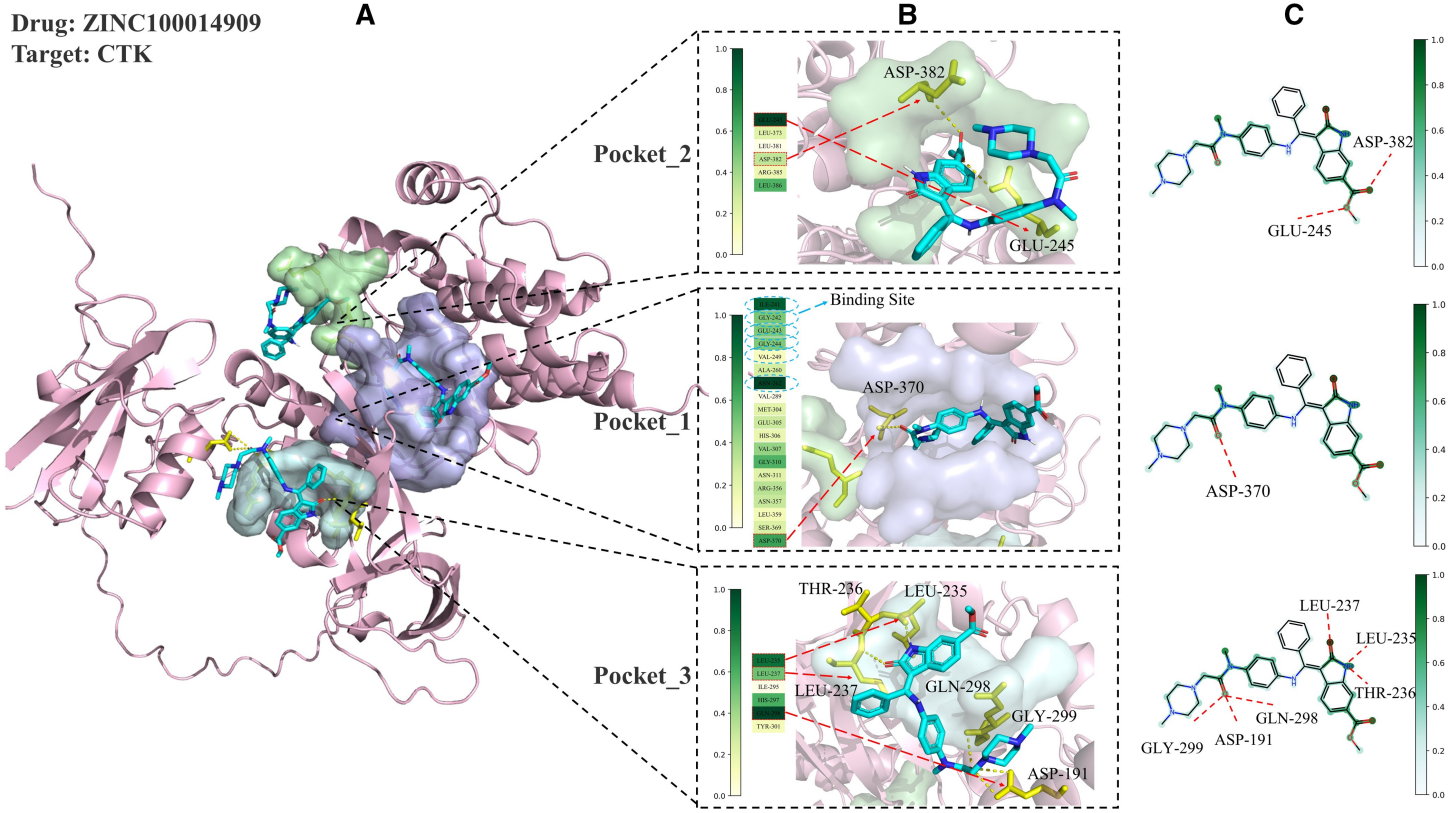
CTK molecular docking and PocketDTA interaction interpretability analysis. (A) Overview of molecular docking complexes: visualizes the complexes formed with ZINC10014909 across three distinct target binding pockets. (B) Interaction interpretability analysis: the left side displays a bilinear attention map where greener shades indicate amino acid residues with higher contributions to DTI; blue-dotted circles mark experimentally validated binding sites. On the right, a 3D close-up highlights amino acid residues involved in hydrogen bonding (in yellow) during docking. (C) Molecular contribution visualization: a 2D plot showing drug atoms' contributions to DTI, with greener shades indicating higher significance and red-dashed lines denoting hydrogen bonds.

Another strength of PocketDTA is the ability to perform interpretable analysis of DTI using the pairwise interaction matrix A. Specifically, through average pooling and normalization operations on the matrix A, we generated an attention weight map of drug heavy atoms and amino acid residues that visualizes their contributions to DTI. In addition, we conducted molecular docking and visualization analyses using QuickVina-W (Hassan *et al.* 2017) and PyMOL 2.5.7 (Schrödinger 2017) tools, and compared the results with the attention weight map. To ensure analytical consistency, molecular docking was performed based on three different target binding pockets (corresponding to the top-3 selected by PocketDTA). We randomly selected a sample from each benchmark dataset for molecular docking comparison analyses. As illustrated in Fig. 7, the molecular docking and weight visualization analyses for the target CTK and the drug ZINC10014909 (Nintedanib) on the Davis dataset are presented. Regarding amino acid residues, Fig. 7B highlights key residues forming hydrogen bonds, which are emphasized in PocketDTA (indicated in a greener hue). Interestingly, significant binding sites such as ILE-241, GLY-242, GLU-243, GLY-244, VAL-249, and ASN-262, as documented in UniProt, received higher emphasis in PocketDTA. Among these, ASN-262 was the primary focus within the target amino acid sequence. This finding clearly demonstrates the advantages of localizing binding pocket information and incorporating 3D structural information. Regarding drug heavy atoms, Fig. 7C shows heavy atoms forming hydrogen bonds with key residues, which are similarly highlighted in green in PocketDTA. Further literature review revealed that the key drug functional groups of interest to PocketDTA, such as amino (–NH_2_), carbonyl (–C = O), and amide groups (–CONH–), align closely with those documented in the literature for Nintedanib’s (Roth *et al.* 2015) possible noncovalent interactions with its targets. This consistency is due to the integration of the drug 3D structural information.

**Figure 8.**
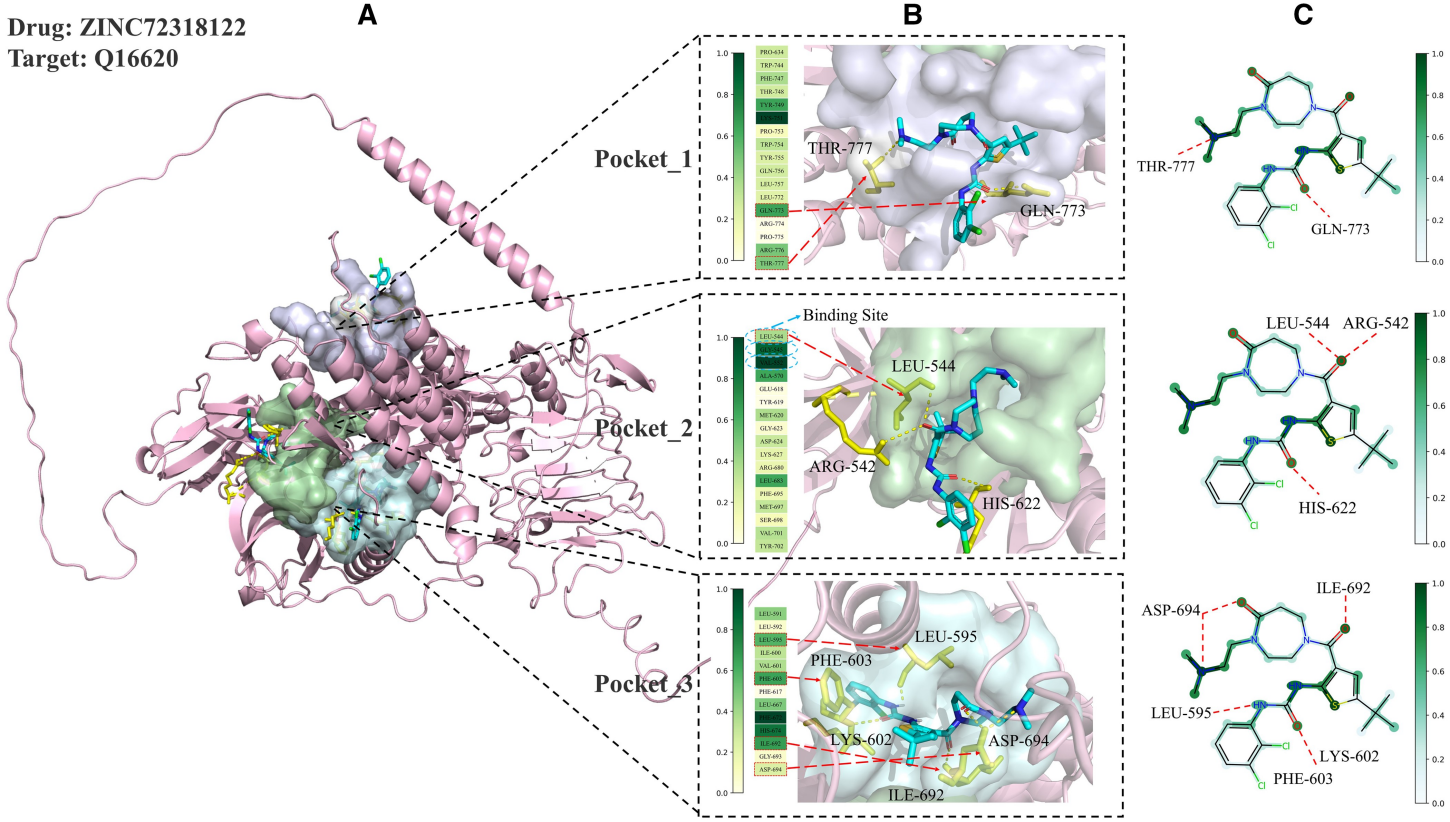
Q16620 molecular docking and PocketDTA interaction interpretability analysis.

Figure 8 depicts target Q16620 with drug ZINC72318122 on the KIBA dataset. Although PocketDTA was able to emphasize key amino acid residues and drug atoms more prominently, it did not perform as well as the Davis dataset in terms of the accuracy of the visualized weights of amino acid residues. In the analysis of the KIBA dataset, some key amino acid residues forming hydrogen bonds with the molecule did not receive significant attention weight. This phenomenon is largely attributed to the small number of target species in the dataset, as also evidenced in Supplementary Figs S3 and S4. However, it is noteworthy that PocketDTA successfully assigned higher weights to UniProt-documented authentic binding sites, such as LEU-544, GLY-545, and VAL-552. Higher weights were also assigned to key molecular functional groups, such as amide groups (–CONH–) and thioether groups (–S–). To minimize bias in the results, we analyzed a larger number of samples (Supplementary Figs S3 and S4). This observation confirms the ability of our model to identify key DTI information and contributes to understanding the potential mechanisms of DTI.

## 5 Conclusion

In this study, we introduced PocketDTA, a new end-to-end method for DTA prediction, with key contributions in dataset quality enhancement, efficient use of 3D structural information, multimodal biological representations, and interpretability of local interactions.

Despite the results achieved in this study, limitations of the model were identified in real-world drug screening applications. Aggregation occurs due to the uneven distribution of affinity values in the training set, which leads to similar aggregation in the real-world prediction results. Therefore, extending and diversifying the DTA benchmark dataset is believed to be key to enhancing the generalization ability of deep learning DTA models. Future research should consider obtaining additional datasets that better meet the practical needs for model evaluation and validation. In addition, plans have been made to explore more flexible training strategies for DTA prediction of new drugs and targets in future studies.

## Author contributions

Long Zhao organized all the data, performed the analyses, designed the study and methodology, conducted the experiments, and drafted the manuscript. Hongmei Wang participated in discussing and revising the manuscript. shaoping Shi supervised the study, revised the manuscript, and approved the final version of the manuscript.

## Supplementary Material

btae594_Supplementary_Data
