# Supplementary material for: PocketDTA: an advanced multimodal architecture for enhanced prediction of drug−target affinity from 3D structural data of target binding pockets
Source: Bioinformatics. 2024 Oct 4;40(10):btae594. doi: 10.1093/bioinformatics/btae594 (PMC11502498; doi:10.1093/bioinformatics/btae594)
Supplement: btae594_Supplementary_Data [file btae594_supplementary_data.pdf]

Supplementary Material

PocketDTA: an advanced multimodal architecture for enhanced prediction of drug-target affinity from 3D structural data of target binding pockets

Long Zhao†, *Hongmei Wang*†, *Shaoping Shi**†

†Department of Mathematics, School of Mathematics and Computer Sciences, Nanchang University, Nanchang, 330031, China, and Institute of Mathematics and Interdisciplinary Sciences, Nanchang University, Nanchang, 330031, China

* To whom correspondence should be addressed: shishaoping@ncu.edu.cn

Contents

1 Materials and methods

1.1 Benchmark datasets

1.2 Data preprocessing

1.3 Target structure representation

1.4 GVP-GNN Layers

1.5 Evaluation metrics

2 Results

2.1 Comparative analysis of performance before and after dataset preprocessing

2.2 Representation ablation study

2.3 Cold start experiment

3 Supplementary Tables

Table S1. Statistics of the benchmarking datasets before and after data processing.

Table S2. Processing methods for the 3D structure of Davis targets.

Table S3. Hyperparameters search range and selected value.

Table S4. Comparative evaluation of various methods for characterizing drug sequences on the benchmark datasets.

Table S5. Comparative evaluation of various methods for characterizing drug structures on the benchmark datasets.

Table S6. Comparative evaluation of various methods for characterizing target sequences on the benchmark datasets.

Table S7. Comparative evaluation of various methods for characterizing target

structures on the benchmark datasets.

Table S8. Comparison of trainable parameters and training time of PocketDTA and SOTA methods on the benchmark datasets.

Table S9. Comparative evaluation of the performance of different models before and after data preprocessing on the benchmark datasets.

Table S10. Results of representation ablation studies on the benchmark datasets.

Table S11. Results of module ablation studies on the benchmark datasets.

Table S12. Performance evaluation on more realistic settings of Davis datasets.

4 Supplementary Figures

Figure S1. Statistical analysis of the Davis and KIBA datasets.

Figure S2. Comparison of the performance of different models before and after data preprocessing on the benchmark datasets.

Figure S3. Visualization and analysis of bilinear attention map weights on the Davis dataset.

Figure S4. Visualization and analysis of bilinear attention map weights on the KIBA dataset.

References

1 Materials and methods

1.1 Benchmark datasets

The Davis dataset includes 68 kinase inhibitors interacting with 442 kinases, covering more than 80% of the human catalytic protein kinome. Its measured dissociation constants (K_d) span from 0.1 nM to 10,000 nM, with lower K_d values indicating higher binding affinity. Typically, K_d values undergo certain mathematical transformations (as shown in equation (1)) to generate affinity scores. The KIBA dataset incorporates different biological activity values, including K_i, K_d, and IC_{50} values, to calculate the KIBA score. The dataset comprises 2068 drugs and 228 kinases, and the KIBA score extends from 0 to 17.2, where higher scores indicate greater binding affinity.

$$pK_d = -\,log_{10}\left(\frac{K_d}{10^9}\right) \tag{1}$$

1.2 Data preprocessing

By analyzing the distribution of affinity values in the two datasets (Figure S1 A), we found that this distribution posed a challenge for model training. Specifically, the distribution of affinity values in the Davis dataset demonstrates significant skewness, primarily concentrated around the value 5. In contrast, the affinity values of the KIBA dataset, although relatively dispersed, still exhibit a tendency towards central clustering.

To optimize the dataset, we undertook the following preprocessing steps: 1) the protein sequences were updated by visiting the UniProt website (updated on January 24, 2024) (Bateman, et al., 2023). In the Davis dataset, targets are identified based on gene names, which means there can be targets with different gene names but identical sequences (e.g., ABL1(E255K) vs. ABL1). The majority of these genes (e.g., AXL,

CSNK2A2, etc.) were updated directly with the corresponding UniProt ID. For mutant targets (e.g., ABL1 (T315I), EGFR (E746A750del), etc.) and domain-specific targets (e.g., TYK2 (JH1 domain-catalytic), RPS6KA4 (KinDom.2-C-terminal), etc.), specific sequence modifications were conducted. However, for phosphorylated targets (e.g., ABL1(E255K)-phosphorylated, ABL1-phosphorylated, etc.), the original sequences were preserved due to the challenges in sequence processing. For the KIBA dataset, whose targets are based on UniProt IDs, the updated protein sequences directly from UniProt. 2) After updating the protein sequences, certain samples remained in both datasets with identical drug SMILES strings and protein sequences, but with inconsistent affinity values, potentially causing interference in model training. Consequently, these noisy samples were deleted to generate the processed benchmark datasets. The statistics of which are presented in Table S1.

Subsequently, the 3D structures of the proteins were acquired. In the Davis dataset, various methods were employed to acquire the 3D structures, depending on the target class (refer to Table S2). For the KIBA dataset, all structures were sourced from the AlphaFold2 protein structure database (version 2022-11-01) (Varadi, et al., 2022) using UniProt ID. AlphaFold2 has demonstrated accuracy in modeling the protein domains of human kinases. Meanwhile, both Davis and KIBA datasets are based on human kinase proteins. Therefore, known and relevant structural domains for kinase activity (e.g., those annotated in UniProt, including protein kinase, PIPK, AGC, etc.) were selected as the initial 3D structures of proteins (Modi and Dunbrack Jr, 2022) to obtain protein 3D structures with higher confidence. AlphaFold2's per-residue confidence score

(pLDDT) ranges from 0 to 100, where higher scores denote higher structural prediction accuracies. Scores above 90 in pLDDT are generally considered to reflect a high degree of confidence, while those below 50 may signify a low-confidence or structure-absent region. In practice, a pLDDT score above 70 is commonly used as a threshold, suggesting that the backbone prediction of the protein structure has a reasonable level of accuracy (Varadi, et al., 2022). Figure S1B and C demonstrate the changes in sequence length and average pLDDT values of proteins before and after domain processing. This step not only effectively reduces the length of protein sequences but also removes a number of residues with low pLDDT values, leading to an increase in the average pLDDT values of most proteins to above 80.

Ultimately, the appropriate cutoffs for protein and drug sequences were determined to prevent representations from becoming sparse due to excessive zero-padding. The 90th percentile values for protein sequence lengths and drug heavy atom counts were selected as thresholds (indicated by red dashed lines in Figure S1B and D).

1.3 Target structure representation

On one hand, most DTIs are facilitated by critical amino acid residues (binding sites) located in target binding pockets (Tubiana, et al., 2022). Numerous prediction tools (Kandel, et al., 2021; Wang, et al., 2022) for target binding pockets have been developed. On the other hand, in molecular docking, the docking box defines the binding region (binding pockets) where a drug potentially interacts with its target. Configuring the docking box narrows the search area, improves docking accuracy, and optimizes energy evaluation. Inspired by the docking box setup, the target binding pockets were predicted

using DoGSite3 (Graef, et al., 2023), a rapid high-precision tool for predicting target binding pockets. DoGSite3 has been open-sourced on the website ProteinsPlus (Schöning-Stierand, et al., 2020) (https://proteins.plus/). The high-confidence 3D structural coordinates of the target (refer to Supplementary Material S1.2) were used as input to obtain the target binding pocket coordinates. DoGSite3 revealed that the first three predicted pockets included the known target binding sites. As a result, the coordinate information of these pockets was retained as model inputs.

Most previous methods for target structure representation often depict the structure as a 2D graph, with residues as nodes and edges defined by spatial distances (He, et al., 2023; Jiang, et al., 2020; Voitsitskyi, et al., 2023). However, since these approaches indirectly capture vector attributes (likes node and edge orientations) by encoding rotationally invariant scalars (likes pairwise distances, angles), they typically fail to adequately capture intricate spatial relationships. To efficiently capture the spatial structure and variations of targets, we represent them using the predicted backbone atoms (C_α, C, N, and O) of each amino acid in the first three pockets 3D coordinates. The target binding pocket is denoted as $\mathcal{G} = (\mathcal{V}, \mathcal{E})$, where each node $v_i \in \mathcal{V}$ represents an amino acid. The set of edges is $\mathcal{E} = \{\varepsilon_{j \to i}\}_{i \neq j}$ encompasses all i, j such that v_j ranks among the $k = 30$ nearest neighbors of v_i, based on the distance between their C_α atoms. Assuming that X_i represents the position of the X atom in the $i\text{-}th$ amino acid (e.g., C_i is the coordinate of the C atom in the $i\text{-}th$ amino acid). The node features and the edge features can be represented respectively as:

Node features: $v_i = (v_i^s, v_i^v)$. The scalar features of each node, denoted as $v_i^s \in$

$\mathbb{R}^{\eta}$ (where η represents the dimension of scalar features), are defined through the calculation of dihedral angles using $N_i, C_{\alpha_i}, C_i, C_{i-1}$ and N_{i+1}, from which sine and cosine values are derived. The vector features of each node, $v_i^v \in \mathbb{R}^{\mu\times 3}$ (where μ denotes the dimension of vector features), comprise unit vectors in the forward and reverse directions of $C_{\alpha_{i+1}} - C_{\alpha_i}$ and $C_{\alpha_{i-1}} - C_{\alpha_i}$, along with the unit vector in the direction of $C_{\beta_i} - C_{\alpha_i}$, collectively defining the orientation of each amino acid residue explicitly. The unit vector in the direction of $C_{\beta_i} - C_{\alpha_i}$ assumes tetrahedral geometry and is computed according to equation (2):

$$\frac{\sqrt{\frac{1}{2}}(n\times c)}{\|n\times c\|_2} - \frac{\sqrt{\frac{2}{3}}(n+c)}{\|n+c\|_2}, \tag{2}$$

where $n = N_i - C_{\alpha_i}$, $c = C_i - C_{\alpha_i}$.

Edge features: $\varepsilon_{j\to i} = \left(\varepsilon_{j\to i}{}^s, \varepsilon_{j\to i}{}^v\right)$. The scalar features of each edge $\varepsilon_{j\to i}{}^s$ are encoded by Gaussian radial basis functions of the distance $\left\|C_{\alpha_j} - C_{\alpha_i}\right\|_2$ and sinusoidal encoding (Vaswani, et al., 2017) of relative position information with scalar characteristics. The vector features of each edge $\varepsilon_{j\to i}{}^v$ are denoted by the unit vector in the direction of $C_{\alpha_j} - C_{\alpha_i}$. Such a constructed representation is sufficient to comprehensively describe the target pockets backbone.

1.4 GVP-GNN Layers

The GVP computational process is as follows:

$$\boldsymbol{V}_h = \boldsymbol{W_h}\boldsymbol{V}, \tag{3}$$

$$s' = \mathrm{ReLU}(\boldsymbol{W_m}Concat(s, \|\boldsymbol{V}_h\|_2) + b), \tag{4}$$

$$\boldsymbol{V}' = \sigma\left(\left\|\boldsymbol{W_\mu}\boldsymbol{V}_h\right\|_2\right) \odot \boldsymbol{W_\mu}\boldsymbol{V}_h, \tag{5}$$

where weight matrices $\boldsymbol{W_h} \in \mathbb{R}^{h\times\mu}$, $\boldsymbol{W_m} \in \mathbb{R}^{\eta'\times(\eta+h)}$, $\boldsymbol{W_\mu} \in \mathbb{R}^{\mu'\times h}$, bias vector $b \in$

$\mathbb{R}^{\eta'}$, $\sigma(\cdot)$ is Sigmoid function, and $\odot$ is inner product. In this process, the GVP concatenates the initial scalar features with the transformed vector features, enabling the model to extract rotationally invariant information from the vector inputs, thereby enhancing information propagation among the nodes in the graph.

1.5 Evaluation metrics

Specifically, MSE serves as a measure of the deviation of the model's predicted values from the observed values. MSE is calculated as follows:

$$MSE = \frac{1}{n}\sum_{i=1}^{n}\left(Y_i - Y_i^{'}\right)^2, \tag{6}$$

where n denotes the number of samples, Y^i is the actual observed value of the i-th sample, and $Y_i^{'}$ is the corresponding predicted value by the model. The smaller the MSE value, the better the model's fit.

CI quantifies the agreement between the rankings of affinity values predicted by the model and those determined experimentally, serving as a criterion for assessing the model's ability to predict rankings during drug screening. A larger CI value indicates a superior model prediction. The CI is calculated using the following formula:

$$CI = \frac{1}{Z}\sum_{\delta_i > \delta_j} h\left(b_i - b_j\right), \tag{7}$$

where b_i is the predicted value of δ_i, b_j is the predicted value of δ_j, Z is the normalization constant and $h(\cdot)$ is the step function:

$$\hbar(x) = \begin{cases} 0, & x < 0 \\ 0.5, & x = 0 \\ 1, & x > 0\,. \end{cases} \tag{8}$$

The r_m^2 index assesses the external prediction performance of the model; ideally, an r_m^2 value close to 1 indicates good external prediction performance and high reliability, as defined below:

$$r_m^2 = r^2(1 - \sqrt{(r^2 - r_0^2)}), \tag{9}$$

where r^2 and r_0^2 are the squared correlation coefficients with and without intercept.

Pearson is used to measure the strength of the linear correlation between the predicted value p and the true value y. The closer the value is to 1, the better the predictive performance of the model, which is defined as follows:

$$Pearson = \frac{\varphi(p,y)}{\varphi(p)\varphi(y)}, \tag{10}$$

where $\phi(p, y)$ is the covariance between the predicted value and the label, $\phi(p)$ is the standard deviation of p, and $\phi(y)$ is the standard deviation of y.

Spearman assesses the dependency between two variables. the value closer to 1 signifies a superior model prediction performance, as defined below:

$$Spearman = 1 - \frac{6\sum d_i^2}{n(n^2-1)}, \tag{11}$$

where d_i denotes the difference between two ranks in the predicted values and labels.

2 Results

2.1 Comparative analysis of performance before and after dataset preprocessing

In S1.2 Data preprocessing, we conducted an optimization process on the benchmark datasets and removed noisy data. To demonstrate the effectiveness of this preprocessing measure, we compared the performance changes between the original and preprocessed benchmark datasets using existing SOTA models. Figure S2 clearly shows that the preprocessing measures enhanced the prediction performance of the models, and the detailed results are presented in Table S9.

2.2 Representation ablation study

A reduction in model performance was observed when the sequence features of both

drugs and targets were excluded from PocketDTA. In particular, the MSE increased by 18.080% (Davis) and 17.857% (KIBA), respectively, which illustrates the crucial role of sequence features in enhancing the model's predictive ability. Moreover, the exclusion of 3D structural features from both drugs and targets severely undermined model performance. The MSE increased by 80.790% (Davis) and 14.286% (KIBA), respectively, confirming the PocketDTA model advantage in integrating 3D structural information of the drug and target. This information complements sequence data, which cannot fully capture 3D interaction details. Additionally, separate ablation experiments for the drug sequence, drug structure, target sequence, and target structure features were carried. Detailed results are shown in Table S10.

2.3 Cold start experiment

In the cold drug strategy, the drugs were divided into sections with an 8:1:1 ratio to ensure that the drugs in the training, validation, and testing sets are distinct from each other. The cold target strategy uses the same segmentation ratio for targets, while the all cold strategy applies this segmentation to both drugs and targets. To minimize experimental error, we repeated the experiment five times, using a different random seed for each run.

3 Supplementary Tables

Table S1. Statistics of the benchmarking datasets before and after data processing.

Dataset	Class	Original	Processed
Davis	Samples	30056	29512
	Drugs	68	68
	Targets	442	434
KIBA	Samples	118083	117395
	Drugs	2068	2061
	Targets	228	228

Table S2. Processing methods for the 3D structure of Davis targets.

Class	Gene Name	3D Structure Processing Methods
No mutated targets	AXL, CSNK2A2, MST4, IRAK3, MUSK, CHEK1 and 368 other targets	Accessed at https://alphafold.ebi.ac.uk/
Mutated targets	ABL1 (T315I), EGFR (E746A750del), GCN2 (KinDom2S808G), KIT (V559D-T670I) and 49 other targets	ESMfold(Lin, et al., 2023)
Targets linked to partial target domains	TYK2(JH1domain-catalytic), TYK2(JH2domain-pseudokinase), RPS6KA4(KinDom.1-N-terminal), RPS6KA4(KinDom.2-C-terminal) and 17 other targets	Intercepted Sequences and AlphaFold Predicted Structures
Phosphorylation targets	ABL1(E255K)-phosphorylated, ABL1-phosphorylated and 6 other targets	Delete
others	PKAC-alpha, PKAC-beta	ESMfold

Table S3. Hyperparameters search range and selected value.

Dataset	Hyperparameter	Search range	Selected Value
Davis	Learning rate	[0.001, 0.002, 0.005, 0.0001, 0.0002, 0.0005]	0.001
	Batch_size	[16, 32, 64, 128, 256, 512]	32
	n_heads	[3, 4, 5, 6, 7, 8]	6
	h_d	[16, 32, 64, 128, 256, 512]	256
	h_t	[64, 128, 256, 512, 1280]	1280
	GVP-GNN layers	[1, 2, 3, 4]	3
	GVP_node_h_dim	[(64, 8), (128, 16), (256, 32), (512, 64)]	(128, 16)
	h_b	[32, 64, 128, 256, 512, 1280]	128
	Weight_decay	[0.0001, 0.0005, 0.001, 0.005]	0.0001
	Lr_decay	[1, 0.95, 0.90, 0.85]	0.95
KIBA	Learning rate	[0.001, 0.002, 0.005, 0.0001, 0.0002, 0.0005]	0.002
	Batch_size	[16, 32, 64, 128, 256, 512]	32
	n_heads	[3, 4, 5, 6, 7, 8]	5
	h_d	[16, 32, 64, 128, 256, 512]	128
	h_t	[64, 128, 256, 512, 1280]	1280
	GVP-GNN layers	[1, 2, 3, 4]	1
	GVP_node_h_dim	[(64, 8), (128, 16), (256, 32), (512, 64)]	(128, 16)
	h_b	[32, 64, 128, 256, 512, 1280]	256
	Weight_decay	[0.0001, 0.0005, 0.001, 0.005]	0.005
	Lr_decay	[1, 0.95, 0.90, 0.85]	0.95

Table S4. Comparative evaluation of various methods for characterizing drug sequences on the benchmark datasets.

Dataset	Drug Features	MSE ↓	CI ↑	r_m^2 ↑	pearson ↑	spearman ↑
Davis	Morgan	**0.177 ± 0.013**	**0.903 ± 0.005**	**0.731 ± 0.017**	**0.887 ± 0.008**	**0.708 ± 0.005**
	Mol2Vec	0.183 ± 0.011	0.899 ± 0.003	0.728 ± 0.012	0.883 ± 0.006	0.701 ± 0.005
	MOLE-BERT	0.180 ± 0.011	0.900 ± 0.003	0.723 ± 0.018	0.885 ± 0.006	0.703 ± 0.006
KIBA	Morgan	**0.140 ± 0.004**	**0.892 ± 0.002**	**0.771 ± 0.011**	**0.896 ± 0.003**	**0.885 ± 0.003**
	Mol2Vec	0.161 ± 0.003	0.868 ± 0.001	0.728 ± 0.008	0.879 ± 0.002	0.858 ± 0.002
	MOLE-BERT	0.144 ± 0.005	0.892 ± 0.002	0.759 ± 0.020	0.893 ± 0.003	0.885 ± 0.002

Bold corresponds to the best performance for each metric, and ↑ / ↓ indicates that the larger/smaller the metrics, the better the model performance.

Table S5. Comparative evaluation of various methods for characterizing drug structures on the benchmark datasets.

Dataset	Drug Features	MSE ↓	CI ↑	r_m^2 ↑	pearson ↑	spearman ↑
Davis	GraphMVP	**0.177 ± 0.013**	**0.903 ± 0.005**	**0.731 ± 0.017**	**0.887 ± 0.008**	**0.708 ± 0.005**
	GraphMVP-C	0.187 ± 0.011	0.900 ± 0.004	0.725 ± 0.034	0.881 ± 0.004	0.703 ± 0.007
	GraphMVP-G	0.182 ± 0.009	0.902 ± 0.004	0.725 ± 0.019	0.884 ± 0.006	0.707 ± 0.004
	3Dinfomax	0.181 ± 0.016	0.900 ± 0.005	0.729 ± 0.028	0.884 ± 0.010	0.704 ± 0.006
KIBA	GraphMVP	**0.140 ± 0.004**	**0.892 ± 0.002**	**0.771 ± 0.011**	**0.896 ± 0.003**	**0.885 ± 0.003**
	GraphMVP-C	0.148 ± 0.007	0.889 ± 0.004	0.762 ± 0.016	0.889 ± 0.005	0.881 ± 0.004
	GraphMVP-G	0.144 ± 0.004	0.890 ± 0.001	0.758 ± 0.019	0.893 ± 0.002	0.882 ± 0.002
	3Dinfomax	0.154 ± 0.005	0.877 ± 0.002	0.750 ± 0.013	0.884 ± 0.004	0.868 ± 0.003

Table S6. Comparative evaluation of various methods for characterizing target sequences on the benchmark datasets.

Dataset	Target Features	MSE ↓	CI ↑	r_m^2 ↑	pearson ↑	spearman ↑
Davis	ESM	0.181 ± 0.013	0.901 ± 0.003	0.717 ± 0.016	0.885 ± 0.005	0.705 ± 0.003
	ProtBert	**0.177 ± 0.013**	**0.903 ± 0.005**	**0.731 ± 0.017**	**0.887 ± 0.008**	**0.708 ± 0.005**
	ProtT5	0.203 ± 0.010	0.891 ± 0.003	0.725 ± 0.012	0.868 ± 0.007	0.691 ± 0.005
KIBA	ESM	**0.140 ± 0.004**	**0.892 ± 0.002**	**0.771 ± 0.011**	**0.896 ± 0.003**	**0.885 ± 0.003**
	ProtBert	0.142 ± 0.005	0.891 ± 0.002	0.764 ± 0.024	0.894 ± 0.003	0.883 ± 0.003
	ProtT5	0.142 ± 0.004	0.890 ± 0.001	0.763 ± 0.009	0.894 ± 0.001	0.883 ± 0.001

Table S7. Comparative evaluation of various methods for characterizing target structures on the benchmark datasets.

Dataset	Target Features	MSE ↓	CI ↑	r_m^2 ↑	pearson ↑	spearman ↑
Davis	Original	0.194 ± 0.012	0.898 ± 0.005	0.692 ± 0.026	0.877 ± 0.009	0.701 ± 0.010
	Processed	**0.177 ± 0.013**	**0.903 ± 0.005**	**0.731 ± 0.017**	**0.887 ± 0.008**	**0.708 ± 0.005**
KIBA	Original	0.147 ± 0.006	0.892 ± 0.002	0.754 ± 0.017	0.891 ± 0.004	0.884 ± 0.004
	Processed	**0.140 ± 0.004**	**0.892 ± 0.002**	**0.771 ± 0.011**	**0.896 ± 0.003**	**0.885 ± 0.003**

Table S8. Comparison of trainable parameters and training time of PocketDTA and SOTA methods on the benchmark datasets.

Dataset	Methods	Trainable parameters	Time per batch
Davis	DeepDTA	1932033	6.297
	GraphDTA	2642689	6.220
	FusionDTA	5584044	45.356
	MGraphDTA	3045461	15.364
	3DProtDTA	8887037	25.325
	NHGNNDTA	4527001	39.987
	MDFDTA	6041535	50.145
	PocketDTA	5420722	36.525
KIBA	DeepDTA	1981185	24.250
	GraphDTA	2642689	7.341
	FusionDTA	5584044	190.365
	MGraphDTA	3045461	61.235
	3DProtDTA	8887037	47.802
	NHGNNDTA	4527001	180.369
	MDFDTA	6041535	210.264
	PocketDTA	5098757	111.018

Table S9. Comparative evaluation of the performance of different models before and after data preprocessing on the benchmark datasets.

Dataset	Methods	MSE ↓	CI ↑	r_m^2 ↑	pearson ↑	spearman ↑
Davis	DeepDTA-	0.269 ± 0.018	0.871 ± 0.008	0.653 ± 0.020	0.815 ± 0.013	0.665 ± 0.007
	DeepDTA	0.238 ± 0.026	0.880 ± 0.010	0.693 ± 0.033	0.843 ± 0.019	0.676 ± 0.016
	GraphDTA-	0.240 ± 0.012	0.879 ± 0.006	0.688 ± 0.016	0.838 ± 0.008	0.677 ± 0.015
	GraphDTA	0.240 ± 0.008	0.880 ± 0.006	0.654 ± 0.015	0.844 ± 0.006	0.675 ± 0.014
	3DProtDTA-	0.197 ± 0.013	0.900 ± 0.006	0.719 ± 0.046	0.873 ± 0.013	0.707 ± 0.009
	3DProtDTA	0.193 ± 0.016	0.900 ± 0.005	0.746 ± 0.021	0.877 ± 0.009	0.705 ± 0.007
	NHGNNDTA-	0.213 ± 0.013	0.893 ± 0.007	0.692 ± 0.025	0.859 ± 0.008	0.696 ± 0.016
	NHGNNDTA	0.179 ± 0.011	0.902 ± 0.003	**0.759 ± 0.016**	0.885 ± 0.005	**0.711 ± 0.014**
	PocketDTA-	0.188 ± 0.010	0.898 ± 0.004	0.695 ± 0.027	0.879 ± 0.006	0.704 ± 0.006
	PocketDTA	**0.177 ± 0.013**	**0.903 ± 0.005**	0.731 ± 0.017	**0.887 ± 0.008**	0.708 ± 0.005
KIBA	DeepDTA-	0.226 ± 0.016	0.834 ± 0.009	0.661 ± 0.013	0.825 ± 0.013	0.805 ± 0.015
	DeepDTA	0.195 ± 0.004	0.850 ± 0.003	0.704 ± 0.010	0.851 ± 0.004	0.830 ± 0.006
	GraphDTA-	0.209 ± 0.002	0.840 ± 0.004	0.695 ± 0.011	0.839 ± 0.003	0.815 ± 0.007
	GraphDTA	0.179 ± 0.005	0.862 ± 0.004	0.731 ± 0.009	0.864 ± 0.005	0.847 ± 0.006
	3DProtDTA-	0.158 ± 0.007	0.879 ± 0.001	0.762 ± 0.011	0.882 ± 0.005	0.872 ± 0.002
	3DProtDTA	0.158 ± 0.005	0.880 ± 0.001	0.762 ± 0.012	0.882 ± 0.003	0.873 ± 0.002
	NHGNNDTA-	0.162 ± 0.006	0.876 ± 0.003	0.746 ± 0.020	0.876 ± 0.005	0.870 ± 0.004
	NHGNNDTA	0.157 ± 0.008	0.879 ± 0.003	0.753 ± 0.021	0.882 ± 0.007	0.870 ± 0.005
	PocketDTA-	0.152 ± 0.003	0.886 ± 0.003	0.742 ± 0.022	0.886 ± 0.003	0.877 ± 0.004
	PocketDTA	**0.140 ± 0.004**	**0.892 ± 0.002**	**0.771 ± 0.011**	**0.896 ± 0.003**	**0.885 ± 0.003**

'-' represents the result of without data preprocessing.

Table S10. Results of representation ablation studies on the benchmark datasets.

Dataset	Drug sequence	Drug structure	Target sequence	Target structure	MSE ↓	CI ↑	r_m^2 ↑	pearson ↑	spearman ↑
Davis	−	√	−	√	0.209 ± 0.011	0.889 ± 0.005	0.716 ± 0.026	0.864 ± 0.006	0.687 ± 0.003
	√	−	√	−	0.320 ± 0.077	0.895 ± 0.003	0.487 ± 0.103	0.834 ± 0.028	0.697 ± 0.007
	−	√	√	√	0.180 ± 0.013	0.900 ± 0.003	0.725 ± 0.018	0.885 ± 0.007	0.704 ± 0.006
	√	−	√	√	0.194 ± 0.012	0.892 ± 0.004	0.692 ± 0.016	0.878 ± 0.008	0.691 ± 0.004
	√	√	−	√	0.209 ± 0.010	0.888 ± 0.007	0.714 ± 0.027	0.864 ± 0.010	0.686 ± 0.008
	√	√	√	−	0.223 ± 0.010	0.900 ± 0.003	0.633 ± 0.025	0.861 ± 0.008	0.705 ± 0.004
	√	√	√	√	**0.177 ± 0.013**	**0.903 ± 0.005**	**0.731 ± 0.017**	**0.887 ± 0.008**	**0.708 ± 0.005**
KIBA	−	√	−	√	0.165 ± 0.006	0.867 ± 0.001	0.729 ± 0.009	0.876 ± 0.004	0.855 ± 0.002
	√	−	√	−	0.160 ± 0.008	0.886 ± 0.004	0.730 ± 0.030	0.881 ± 0.005	0.877 ± 0.006
	−	√	√	√	0.164 ± 0.007	0.869 ± 0.001	0.710 ± 0.015	0.878 ± 0.006	0.858 ± 0.002
	√	−	√	√	0.154 ± 0.006	0.888 ± 0.002	0.748 ± 0.024	0.885 ± 0.003	0.879 ± 0.002
	√	√	−	√	0.145 ± 0.006	0.889 ± 0.002	0.763 ± 0.018	0.892 ± 0.004	0.881 ± 0.003
	√	√	√	−	0.154 ± 0.007	0.886 ± 0.002	0.757 ± 0.010	0.884 ± 0.004	0.877 ± 0.003
	√	√	√	√	**0.140 ± 0.004**	**0.892 ± 0.002**	**0.771 ± 0.011**	**0.896 ± 0.003**	**0.885 ± 0.003**

Table S11. Results of module ablation studies on the benchmark datasets.

Dataset	Model	Drug structure Decoder	Drug Target Fusion	MSE ↓	CI ↑	r_m^2 ↑	pearson ↑	spearman ↑
Davis	Model-1	GraphMVP Decoder	Concatenation	0.194 ± 0.019	0.894 ± 0.005	**0.747 ± 0.028**	0.874 ± 0.011	0.694 ± 0.003
	Model-2	FCN	BAN	0.186 ± 0.014	0.896 ± 0.008	0.722 ± 0.015	0.881 ± 0.012	0.696 ± 0.010
	PocketDTA	GraphMVP Decoder	BAN	**0.177 ± 0.013**	**0.903 ± 0.005**	0.731 ± 0.017	**0.887 ± 0.008**	**0.708 ± 0.005**
KIBA	Model-1	GraphMVP Decoder	Concatenation	0.159 ± 0.014	0.882 ± 0.008	0.757 ± 0.019	0.880 ± 0.010	0.874 ± 0.009
	Model-2	FCN	BAN	0.142 ± 0.004	0.890 ± 0.002	**0.782 ± 0.011**	0.893 ± 0.003	0.882 ± 0.002
	PocketDTA	GraphMVP Decoder	BAN	**0.140 ± 0.004**	**0.892 ± 0.002**	0.771 ± 0.011	**0.896 ± 0.003**	**0.885 ± 0.003**

Table S12. Performance evaluation on more realistic settings of Davis datasets.

Setting	Methods	MSE ↓	CI ↑	r_m^2 ↑	pearson ↑	spearman ↑
Cold drug	DeepDTA	0.686 ± 0.167	0.643 ± 0.108	0.156 ± 0.070	0.399 ± 0.096	0.269 ± 0.195
	GraphDTA	0.656 ± 0.267	0.686 ± 0.050	0.196 ± 0.073	0.452 ± 0.093	0.339 ± 0.103
	3DProtDTA	0.651 ± 0.207	0.682 ± 0.044	0.205 ± 0.065	0.475 ± 0.055	0.333 ± 0.106
	NHGNNDTA	0.661 ± 0.196	0.668 ± 0.071	0.166 ± 0.056	0.426 ± 0.063	0.309 ± 0.142
	PocketDTA	**0.531 ± 0.179**	**0.737 ± 0.029**	**0.285 ± 0.037**	**0.550 ± 0.029**	**0.426 ± 0.068**
Cold target	DeepDTA	0.444 ± 0.077	0.812 ± 0.029	0.420 ± 0.109	0.664 ± 0.098	0.568 ± 0.046
	GraphDTA	0.601 ± 0.039	0.727 ± 0.023	0.254 ± 0.047	0.553 ± 0.054	0.418 ± 0.037
	3DProtDTA	0.358 ± 0.023	0.834 ± 0.011	0.493 ± 0.022	0.756 ± 0.012	0.604 ± 0.019
	NHGNNDTA	0.347 ± 0.027	0.843 ± 0.016	0.530 ± 0.039	0.760 ± 0.030	0.618 ± 0.028
	PocketDTA	**0.316 ± 0.038**	**0.858 ± 0.011**	**0.552 ± 0.032**	**0.788 ± 0.022**	**0.643 ± 0.018**
All cold	DeepDTA	0.790 ± 0.264	0.549 ± 0.112	0.096 ± 0.096	0.258 ± 0.198	0.105 ± 0.218
	GraphDTA	0.942 ± 0.356	0.552 ± 0.047	0.056 ± 0.042	0.236 ± 0.086	0.098 ± 0.083
	3DProtDTA	0.740 ± 0.272	0.675 ± 0.043	0.180 ± 0.065	0.437 ± 0.076	0.317 ± 0.097
	NHGNNDTA	0.738 ± 0.223	0.643 ± 0.055	0.128 ± 0.078	0.359 ± 0.135	0.266 ± 0.122
	PocketDTA	**0.675 ± 0.275**	**0.681 ± 0.082**	**0.199 ± 0.089**	**0.442 ± 0.120**	**0.330 ± 0.157**

4 Supplementary Figures

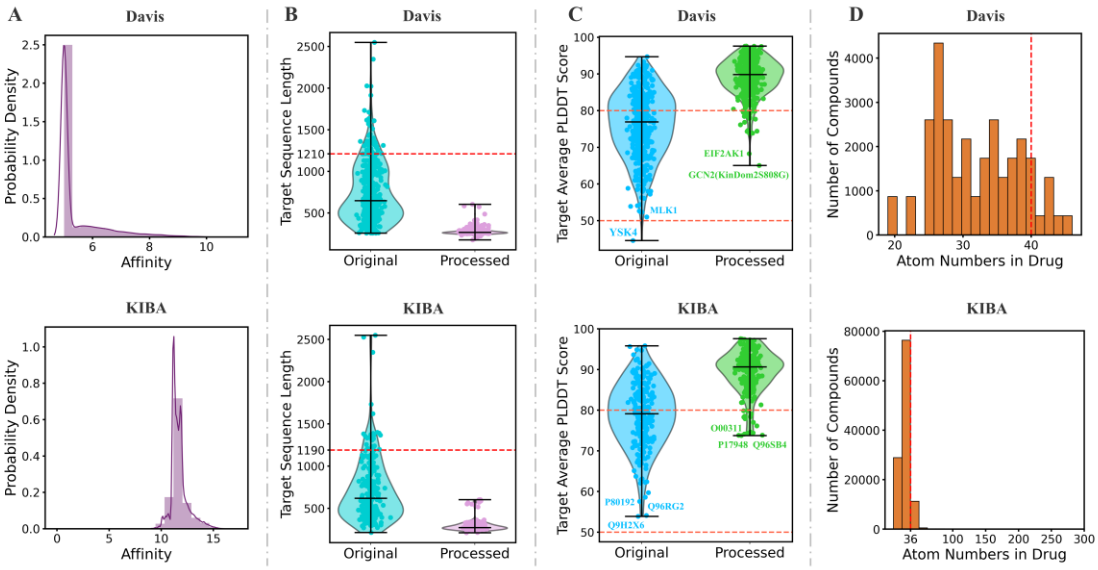

Figure S1. Statistical analysis of the Davis and KIBA datasets. (A) The distribution of affinity values. (B) Comparative analysis of target sequence length before and after data processing. (C) Comparative analysis of mean PLDDT values of targets before and after data processing. (D) Distribution of atomic numbers in drugs.

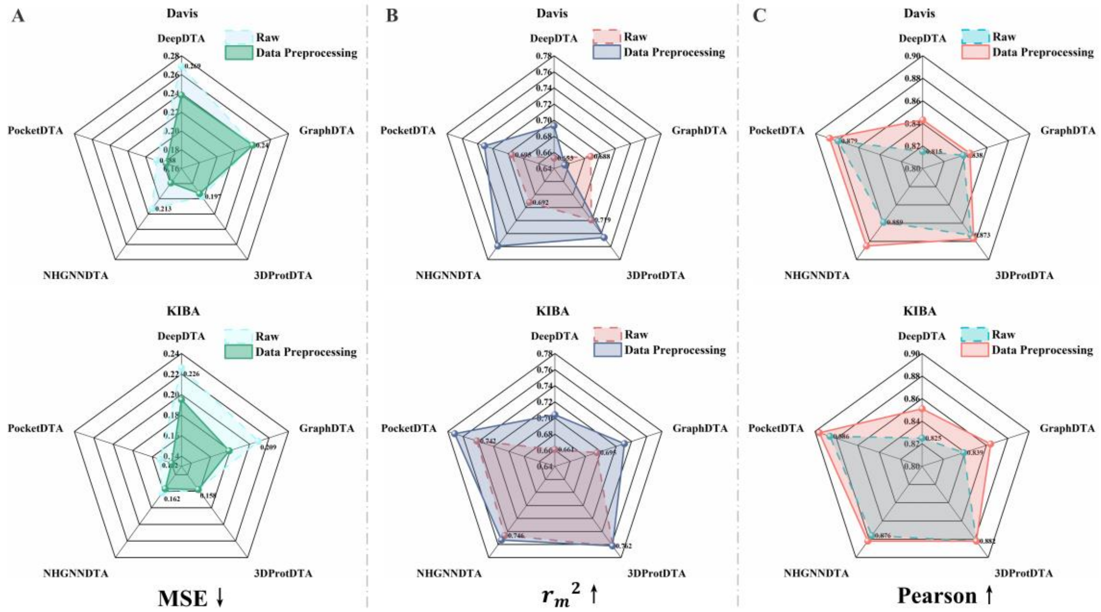

Figure S2. Comparison of the performance of different models before and after data preprocessing on the benchmark datasets.

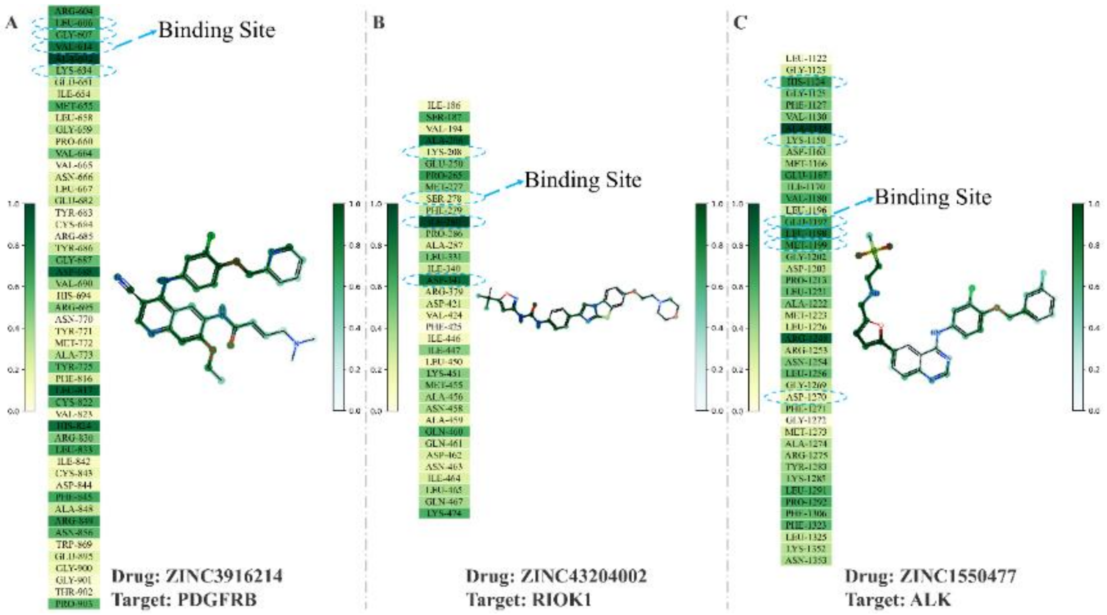

Figure S3. Visualization and analysis of bilinear attention map weights on the Davis dataset.

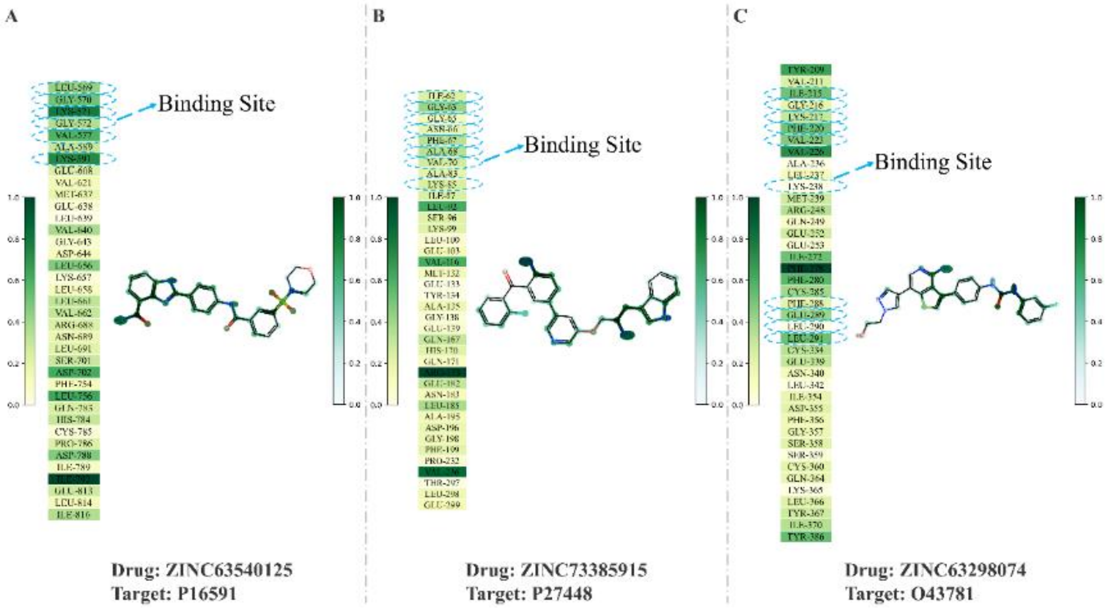

Figure S4. Visualization and analysis of bilinear attention map weights on the KIBA dataset.

References

Bateman, A., *et al.* UniProt: the universal protein knowledgebase in 2023. *Nucleic Acids Res.* 2023;51(D1):D523-D531.

Graef, J., Ehrt, C. and Rarey, M. Binding site detection remastered: enabling fast, robust, and reliable binding site detection and descriptor calculation with DoGSite3. *Journal of Chemical Information Modeling* 2023;63(10):3128-3137.

He, H., Chen, G. and Chen, C.Y.-C. NHGNN-DTA: a node-adaptive hybrid graph neural network for interpretable drug–target binding affinity prediction. *Bioinformatics* 2023;39(6):btad355.

Jiang, M., *et al.* Drug–target affinity prediction using graph neural network and contact maps. *RSC advances* 2020;10(35):20701-20712.

Kandel, J., Tayara, H. and Chong, K.T. PUResNet: prediction of protein-ligand binding sites using deep residual neural network. *Journal of cheminformatics* 2021;13:1-14.

Lin, Z.M., *et al.* Evolutionary-scale prediction of atomic-level protein structure with a language model. *Science* 2023;379(6637):1123-1130.

Modi, V. and Dunbrack Jr, R.L. Kincore: a web resource for structural classification of protein kinases and their inhibitors. *Nucleic Acids Res.* 2022;50(D1):D654-D664.

Schöning-Stierand, K., *et al.* Proteins Plus: interactive analysis of protein–ligand binding interfaces. *Nucleic Acids Res.* 2020;48(W1):W48-W53.

Tubiana, J., Schneidman-Duhovny, D. and Wolfson, H.J. ScanNet: an interpretable geometric deep learning model for structure-based protein binding site prediction. *Nature Methods* 2022;19(6):730-739.

Varadi, M., *et al.* AlphaFold Protein Structure Database: massively expanding the structural coverage of protein-sequence space with high-accuracy models. *Nucleic Acids Res.* 2022;50(D1):D439-D444.

Vaswani, A., *et al.* Attention is all you need. *Advances in neural information processing systems* 2017;30.

Voitsitskyi, T., *et al.* 3DProtDTA: a deep learning model for drug-target affinity prediction based on residue-level protein graphs. *RSC advances* 2023;13(15):10261-10272.

Wang, X., *et al.* DUnet: A deep learning guided protein-ligand binding pocket prediction. *bioRxiv* 2022:2022.2008. 2011.503579.
